# Exercising with Baxter: preliminary support for assistive social-physical human-robot interaction

**DOI:** 10.1186/s12984-020-0642-5

**Published:** 2020-02-17

**Authors:** Naomi T. Fitter, Mayumi Mohan, Katherine J. Kuchenbecker, Michelle J. Johnson

**Affiliations:** 1 Collaborative Robotics and Intelligent Systems Institute, Oregon State University, Corvallis, OR USA; 2 Department of Mechanical Engineering and Applied Mechanics, University of Pennsylvania, Philadelphia, PA USA; 3 Haptic Intelligence Department, Max Planck Institute for Intelligent Systems, Stuttgart, Germany; 4 Department of Physical Medicine and Rehabilitation, University of Pennsylvania, Philadelphia, PA USA

**Keywords:** Socially assistive robotics, Physical human-robot interaction, Exercise games, Personal robots

## Abstract

**Background:**

The worldwide population of older adults will soon exceed the capacity of assisted living facilities. Accordingly, we aim to understand whether appropriately designed robots could help older adults stay active at home.

**Methods:**

Building on related literature as well as guidance from experts in game design, rehabilitation, and physical and occupational therapy, we developed eight human-robot exercise games for the Baxter Research Robot, six of which involve physical human-robot contact. After extensive iteration, these games were tested in an exploratory user study including 20 younger adult and 20 older adult users.

**Results:**

Only socially and physically interactive games fell in the highest ranges for pleasantness, enjoyment, engagement, cognitive challenge, and energy level. Our games successfully spanned three different physical, cognitive, and temporal challenge levels. User trust and confidence in Baxter increased significantly between pre- and post-study assessments. Older adults experienced higher exercise, energy, and engagement levels than younger adults, and women rated the robot more highly than men on several survey questions.

**Conclusions:**

The results indicate that social-physical exercise with a robot is more pleasant, enjoyable, engaging, cognitively challenging, and energetic than similar interactions that lack physical touch. In addition to this main finding, researchers working in similar areas can build on our design practices, our open-source resources, and the age-group and gender differences that we found.

## Background

Increases in life expectancy foreshadow the need for more accessible healthcare solutions in the United States and beyond [1, 2]. Society will soon encounter limits not only on the capacity of assisted living facilities, but also on medical services at large. Thus, solutions that bolster the health of older adults while allowing them to live independently will become more important. One strategy to enhance our society’s ability to keep older adults **healthy and active** in their homes is the introduction of assistive robots in everyday environments.

**Fig. 1:**
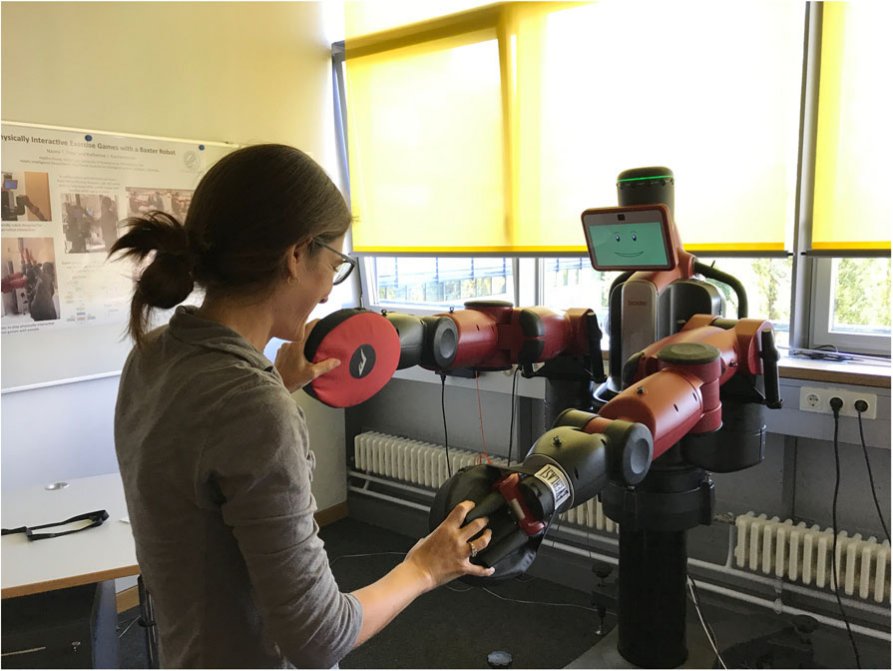
Example human-robot exercise game interaction. Our customization of this robot’s facial expressions and end-effectors help Baxter serve as a gameplay partner

A key contribution of this work is understanding how robots with **social interaction skills***and***dynamic physical interaction skills** can *encourage exercise*. Generally, low-impact exercises are recommended to keep older individuals cognitively and physically well [3–5]. Researchers have already found that robotic exoskeletons can promote upper-limb exercise by physically interacting with human users [6]. Other investigations have indicated that robots can motivate older adults to stay active via social exercise encouragement [7, 8]. As pictured in Fig. 1, the exercise games we designed fit at the **new intersection** of physical human-robot interaction and socially assistive robotics. *Our central goal is to determine whether and how a robot can encourage enjoyable light exercise via social-physical exercise games.*

This research builds on our previous investigations of playful hand-clapping robots [9, 10] by applying similar hardware, robot motion, and hand-contact detection strategies to accomplish a wider variety of physical human-robot interactions. Our work explores the use of the Rethink Robotics Baxter Research Robot to promote exercise via eight games, six of which involve dynamic physical human-robot interaction (pHRI). These activities were further designed for personalization to the physical and cognitive abilities of the user. Initial prototypes of these six pHRI games were described in [11] and demonstrated at the 2017 ACM/IEEE International Conference on Human-Robot Interaction [12].

After we review the related work in the next subsection, the Human-Robot Exercise Game Design Section of this paper outlines our game design iteration steps. The Exploratory User Study Methods Section describes the proof-of-concept study that helped us judge the viability of using these games to engage older adults and promote exercise. The Results Section demonstrates that both younger and older people are willing to interact with the robot in playful exercise games and that only physically interactive games fell in the highest ranges for pleasantness, enjoyment, engagement, cognitive challenge, and energy level. The Discussion Section reviews the key points and future directions related to this work.

### Related work

One of the key contributions of our present work is the investigation of exercise games that are both **socially interactive** and **dynamically physically interactive**. *Although each characteristic has been explored in isolation, almost no socially assistive robots make physical contact with people, especially not in a dynamic and high-energy way.* Touch is an essential pathway for human connection and emotion [13], and we thus expect the incorporation of direct physical interaction into social robotic systems to lead to increased engagement and enjoyment. Physical interaction with the hands is of particular interest because it greatly aids human understanding and serves as a channel for complex sensation and expression [14]. A few past projects began to combine social and physical human-robot interaction. The Haptic Creature Project, for example, explores an expressively actuated furry robotic companion that people treat in pet-like ways [15]. The Paro [16], Huggable [17], and HuggieBot [18] robots leverage physical interaction with people to comfort and support them. Our work explores a **new and highly dynamic application of social touch** with the potential to benefit the lives of older adults.

While designing our dynamic social-physical human-robot exercise games, we considered past physical rehabilitation and exercise interventions involving hand contact and the use of assistive robots. Boxing is one hand-to-hand exercise strategy that has been used to help treat cerebral palsy and Parkinson’s Disease [19, 20]. Low-impact exercises are generally recommended to keep people cognitively and physically well [3]. A range of approaches can help facilitate this activity, such as robot exoskeletons [21, 22] and robot end-effectors [23] that promote upper-limb exercise by physically interacting with human users, robots that use social interaction to motivate a user to exercise [7, 8], and systems that encourage gamified arm motion by people with stroke [24]. Music creation has additionally been used as a motivating factor in rehabilitation robotics [25], and a past need-finding study with older adults found that the ability to play games, present music, and promote physical activity are among key design criteria for a robot in an assisted living community [26]. Accordingly, investigations of robot play activities like dancing [27], hugging [28], and performing magic [29] inform our interaction design and evaluation. The related work overall demonstrates that assistive robots using various combinations of social abilities, physical interaction abilities, and playful interaction premises can have distinct benefits for diverse groups of people, from offering enjoyable exercise to individuals with cerebral palsy to improving the well-being of older adults. We intend to **better understand and leverage the unique advantages at the intersection of physical human-robot interaction and socially assistive robotics** for human-robot exercise interactions.

Our efforts were further influenced by past research on robotic assistants for older adult care. A survey of robots that support the independent living of older adults found that there has been a rapid evolution of technology in this area, and that most studies to date have been conducted in laboratories and hospital settings [30]. Another review paper found evidence that socially assistive robots can enhance the well-being of older adults while reducing the workload for caregivers [31], and other work suggests that older adults can adopt new technologies to support better diet and physical activity practices given proper training and guidance [32]. Past ethnographic research provides best practices for introducing a robot into an assisted living facility while also presenting evidence that older adults accepted the robot into the community [33]. Older adults on the whole are not more accepting of robots than younger adults, and some of the negative perceptions held by this age group are similar to those of the younger community [34]. At the same time, past work has found younger and older adult users to uniformly prefer exercise games with a physical robot, compared to an onscreen image of a robot [35, 36]. Older adults and stakeholders in older adult care also value designing robots to center on autonomy and resilience, not just potential challenges associated with aging [37]. Thus, it is important to take care when designing robots for older adults and validate proposed interactions before introducing them in broader communities.

## Human-robot exercise game design

To begin prototyping social-physical human-robot interactions for assistive applications, we needed to identify robotic hardware that is safe for physical interaction, consult with experts, and create a set of games that attempt to motivate different kinds of exercise.

### Hardware for exercise interactions

Based on our past work identifying the Rethink Robotics Baxter Research Robot as a capable platform for social-physical interaction with people [9, 10], we selected Baxter for the present investigation. This robot offers advantages for pHRI and exercise interactions because it is human-sized, anthropomorphic, and safe for physically interactive tasks. Baxter’s mechanical safety features include series elastic actuators, fully backdrivable joints, and impact-absorbing shells. The humanoid anatomy of this robot allows for an intuitive mapping of game motions to the human body, and it was available at the time at a relatively low price (∼$32,000).

Baxter’s commercially available end-effectors proved unsuitable for our envisioned human-robot interactions. Instead, we used Everlast brand boxing pads that were easily placed over the standard parallel-jaw grippers as end-effectors. These lightweight pads allow users to interact quite forcefully with Baxter without pain or discomfort.

External computer speakers were also incorporated into the system to add music and other sounds to game interactions. We used the Mingus synthesizer (a wrapper for the FluidSynth MIDI sound synthesizer) to compose, load, and play musical effects in the exercise games. FluidSynth requires a sound font file; we selected the OmegaGMGS2 sound font, which suited our purposes for playing different notes in various instrument modes.

### Gameplay design

**Fig. 2:**
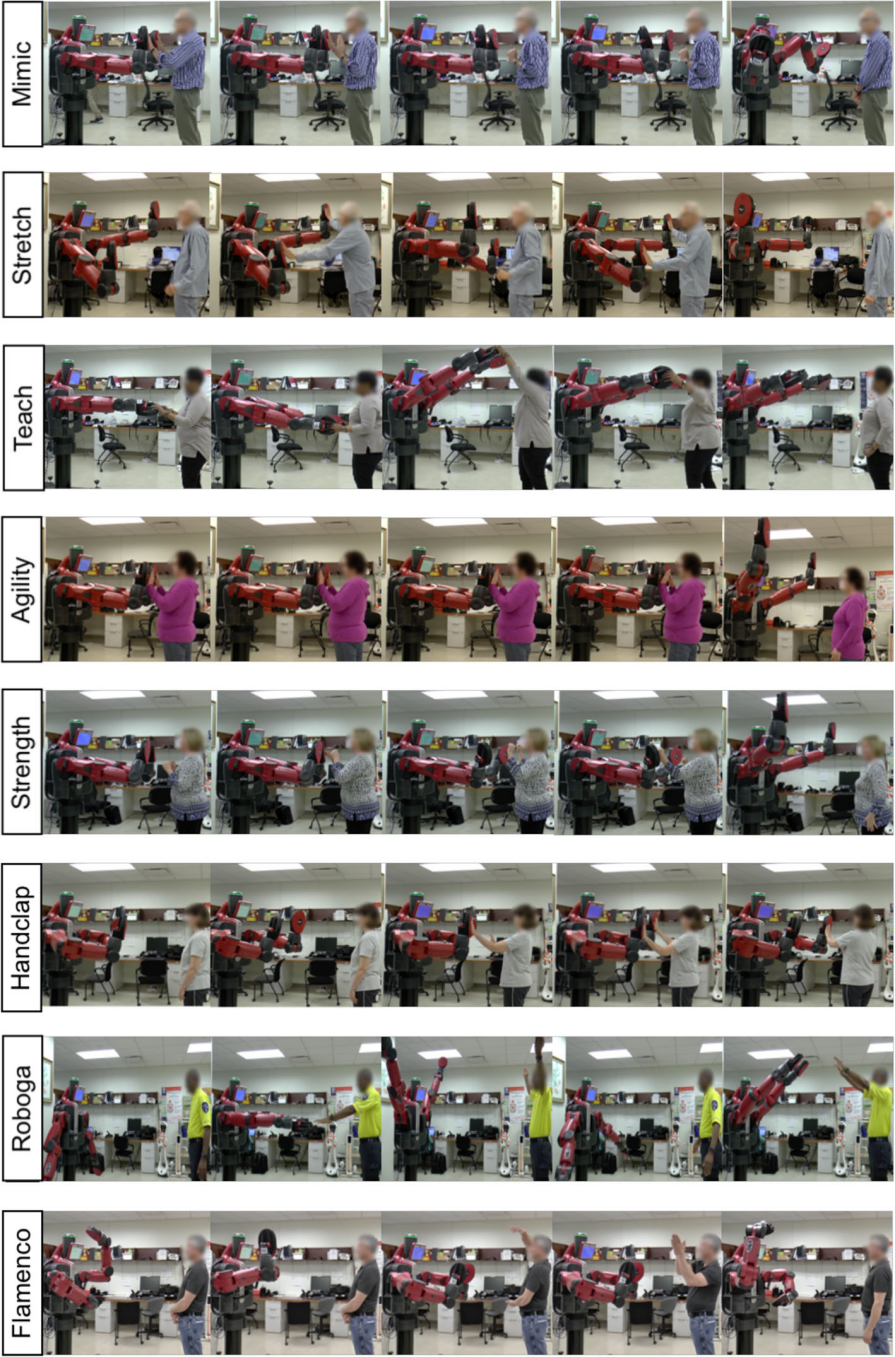
Illustrative frames from the eight developed exercise games
- In the **Stretch Game**, Baxter strikes a series of poses, and the user must mimic its pose and hit its end-effectors in each new pose.
- In the **Teach Game**, the user can move Baxter’s arms to different positions to play and record musical chords mapped to its workspace.
- The **Agility Game** challenges users to wake a “sleeping” Baxter by making rapid contact with its end-effectors.
- The **Strength Game** is a boxing training-like interaction during which Baxter strikes a series of poses and prompts the user to contact its end-effectors.
- In the **Handclap Game**, Baxter teaches the user a sequence of hand-clapping game motions and the user plays the game with the robot.
- The **Roboga Game** (an abbreviated spelling of “Robot Yoga”) requires the user to match and hold stretching poses demonstrated by Baxter.
- In the **Flamenco Game**, Baxter teaches the user a sequence of dance moves to music and the user replicates the dance along with the same music clip.

We designed eight games for users to play with Baxter: the Mimic, Stretch, Teach, Agility, Strength, Handclap, Roboga, and Flamenco Games shown in Fig. 2. Our intention was to create safe and entertaining interactions that promote upper-limb movement while inducing a moderate level of physical and cognitive exercise. To promote social engagement, the games were augmented by a suite of facial expressions [38] and nonverbal behaviors (blinking, changes in emotion, head movements, etc.) implemented using Baxter’s LCD screen and head joints. Music and audiovisual feedback were incorporated into many of the games in an effort to enhance motivation. Concise descriptions of each game follow:

- The **Mimic Game** is a “Simon Says”-style game during which the user teaches Baxter a pattern of left-, right-, and both-handed claps.

**Fig. 3:**
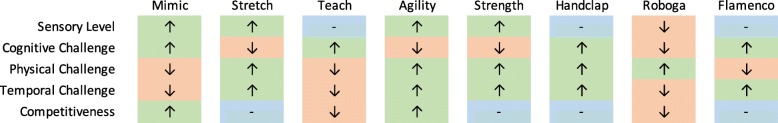
Intended game characteristics of each human-robot exercise game. Upward-facing arrows indicate more emergence of that row’s attribute, downward-facing arrows indicate less emergence, and dashes represent areas where we expected the game to be neutral on the given axis


Games varied across active, passive, or zero physical contact, as well as constant, intermittent, and zero sound. We list the corresponding overall expected sensory level of each game in Fig. 3. We further intended the designs to vary between lower or higher cognitive demand, lower or higher physical demand, lower or higher temporal demand, and competitive or cooperative premises, as summarized in Fig. 3 and described in more detail in Appendix Appendix A: Exercise Game Descriptions and the supplementary materials (Additional files 1, 2, 3, 4, 5, 6, 7, 8, 9, 10, 11, 12, 13, 14, 15, 16, and 17). This diversity of game characteristics allows us to consider how these factors affect user interaction experience.

Additional file 2: Video demonstration of the Mimic Game.



Additional file 4: Video demonstration of the Stretch Game.



Additional file 6: Video demonstration of the Teach Game.



Additional file 8: Video demonstration of the Agility Game.



Additional file 10Video demonstration of the Strength Game.



Additional file 12Video demonstration of the Handclap Game.



Additional file 14Video demonstration of the Roboga Game.



Additional file 16Video demonstration of the Flamenco Game.



Additional file 17Video demonstration and instructions for the eight exercise games.


This work was informed by expert guidance from a game designer, a physical therapist, and an occupational therapist. Feedback throughout iterative design steps with these experts helped us to instill the games with clear social cues by Baxter, adaptive cognitive and physical difficulty levels, and fitting musical premises. These ideas are well supported by past work on designing effective exercise games for therapeutic interactions [39–41]. Throughout the games, the maximum hand-to-hand span of the robot was limited to the user’s height. For games with action speed requirements, we incrementally lowered the speed threshold for users with limited arm motion speed. Games with memory requirements were adjusted based on cognitive ability levels.

## Exploratory user study methods

We conducted an exploratory user study to evaluate how people respond to prompts to play exercise games with Baxter and how such games may fit into assistive applications. Eligible participants played a sample segment of each game, immediately reported their perceptions of that game, and selected their favorite game to try again in a longer free-play interaction. The University of Pennsylvania (Penn) IRB approved all study procedures under protocol 826370.

### Study factors and covariates

This experiment employed a within-subjects design that enabled all participants to experience all eight exercise games pictured in Fig. 2. The experimenter read scripted instructions to each participant to prepare them for each semi-randomly ordered game interaction. When referring to each game, the experimenter used only a letter label (A-H), rather than the game name, to avoid influencing participants’ interaction styles. The between-subjects factor in this study was age; one younger adult and one older adult group of participants completed the study. We sought gender balance in each group to better perform later analyses with gender as a covariate.

### Participants

We recruited participants using flyers in the Philadelphia area and emails to university listservs. Thirty-nine participants (20 male and 19 female) enrolled, gave informed consent, and successfully completed the study. One additional male participant enrolled in the study but broke one of Baxter’s parts and thus did not complete the full study. His partial survey data were excluded from analysis. Participants were divided into two groups: a younger group from 18 to 36 years old (10 male, 10 female, aged 23.6 ± 4.1 years) and an older group from 54 to 70 years old (11 male, 9 female, aged 59.6 ± 3.9 years), where our notation represents the mean ± the standard deviation. All younger adult participants and eight older adults were affiliated with Penn. According to the demographic survey responses, the younger group was made up of seventeen technically trained (for example, working or studying in science, mathematics, engineering, or technology fields) and three non-technical individuals, while the older adult group comprised four technical and fifteen non-technical individuals. On a scale of 0 (least experience) to 100 (most experience), younger participants reported moderate experience levels with robots on average (55.2 ± 25.5) and low experience with Baxter (26.4 ± 19.2). Older adults reported very low experience with robots on average (12.2 ± 14.9) and similarly low experience with Baxter (15.7 ± 19.8). All participants possessed full function in their arms and hands and had normal or corrected-to-normal vision and hearing.

### Pre-study assessment data

To gain some understanding of participant physical and cognitive abilities, we measured each person’s dexterous manipulation abilities with the Box and Blocks manual dexterity assessment (BnB) [42], depression levels with Beck’s Depression Inventory (BDI) [43], and (for older adults) cognitive abilities with the Montreal Cognitive Assessment (MoCA) [44]. We also recorded user height to ensure that the study activities stayed within the physical armspan of the user.

The BnB activity let us confirm that participants had full function in their arms and hands, and it also gave us an idea of their motion speed capabilities. Small adjustments to exercise game timeout periods were made based on the BnB scores (60.21 ± 8.27 blocks moved in one minute), our proxy for participant motion speed. A t-test on BnB score across age group revealed that younger adults (63.3 ± 7.4) moved significantly more blocks than older adults (56.7 ± 8.0) (*p* < 0.001).

Since depression has been shown to influence the activity motivation of depressed individuals, BDI scores (4.02 ± 5.29) were recorded. Two younger adults were in the mild clinical disturbance range (scoring 11 and 13), one younger adult had borderline clinical depression (scoring 17), and one older adult had moderate depression (scoring 25). All other scores were below 11, a range not indicative of any depression or mood disturbance. Younger (4.5 ± 4.6) and older (3.7 ± 6.1) adult BDI scores were not significantly different.

We assumed that younger adult participants were cognitively well, but we administered the MoCA to older adult participants to quantify their cognitive function. The MoCA scores (26.20 ± 2.59) were used to set the Mimic Game sequence length. Six older adult participants scored in the MoCA range indicative of mild cognitive impairment.

### Measurement

Our study software recorded the accelerometer data from Baxter’s onboard wrist accelerometers, the key sensors used to accomplish the logical flow of most of our exercise games. The study was additionally videotaped for later annotation. We asked participants to complete four types of surveys:

- **Survey 1**: a robot evaluation after hearing introductory information about Baxter
- **Survey 2**: an exercise game survey after each gameplay experience
- **Survey 3**: a concluding survey after the free play interaction
- **Survey 4**: a basic demographic survey after the concluding survey

Table 1 summarizes the overall types of collected data. More information about what measures each survey entailed appears below and in the supplementary materials (Additional files 18, 19, 20, and 21).

**Table 1:** Self-reported, annotated, and sensor metrics that helped us understand participant experiences during the exercise games

Source of data	All games	Subset of games
Self-Reports	Exercise level	
	Pleasure	
	Energy level	
	Dominance	
	Pain	
	Safety	
	Enjoyment	
	Engagement	
	Human performance	
	Robot performance	
	Rushedness	
	Calmness	
Video Annotations	Number of tries	
	Task clarifications	
Sensor Recordings		Contact acceleration (Mimic, Stretch, Agility, Strength, Handclap)
		Arc length traveled (Teach)

Surveys 1 and 3 included questions adapted from the Unified Theory of Acceptance and Use of Technology (UTAUT) and other metrics employed in [45] and [46]. The question topic groupings on this survey included:

- attitude toward technology (ATECH, 3 questions)
- cultural context (CC, 3 questions)
- effort expectancy (EE, 2 questions)
- forms of grouping (GR, 3 questions)
- performance expectancy (PE, 3 questions)
- reciprocity (REC, 2 questions)
- self-efficacy from UTAUT model (SE, 4 questions)
- attachment (ATT, 2 questions)

Survey 2 used questions adapted from a variety of relevant sources, as detailed in the list below. Participants responded to each question on a slider scale from 0 to 100.

- Pleasure, energy, and dominance levels: derived from Self-Assessment Manikin (SAM) [47] scales with the standard visual aids representing pleasure, energy, and dominance levels, 3 questions.
- Performance and temporal demand levels: derived from the NASA Task Load Index (TLX) [48] ratings of human performance, robot performance, rushedness (inverse scale), and calmness levels, 4 questions.
- Enjoyability and engagement levels: derived from interaction enjoyment and engagement survey questions used in [49], 2 questions.
- Exercise level: derived from the Borg perceived exertion scale [50], 1 question.
- Pain level: gathered as another perspective on exercise level/muscle burn, derived from the Wong-Baker FACES pain rating scale [51], 1 question.
- Safety level: derived from a scale used in our past work to track user feelings of safety [10], 1 question.

Surveys 2 and 3 also included free response questions to help elicit additional experiential data from users about the following items: enjoyable aspects of the interaction, challenging aspects of the interaction, what stood out overall, and other activities they would like to do with the robot. Survey 4 gathered information about participant age, gender, handedness, profession, technical or non-technical background, experience level with robots, experience level with Baxter, and hometown.

### Study procedure

Each person came to the lab for a single 90-minute session. Before the study interactions began, the participant completed the screening activities mentioned previously: the BnB, BDI, and MoCA. Baxter then waved hello to the user, and the research assistant read a script to relay relevant background information on Baxter. This information was followed by an opening survey about user perception of Baxter (survey 1). Next, the participant stood facing Baxter and played 90-second-long samples of the eight different exercise games in a unique pre-determined order that was balanced across participants. After each exercise game, the user completed a survey about that game (survey 2). The relayed instructions for each game and general gameplay concept for each game are further explained in a video available at [52], and the source code for these exercise games is available at [53]. After the eight games, the user refreshed their memory of the game options by watching video snippets of all the games (in the same order as they experienced the games), selected their favorite game, and entered a free play mode during which they could play that game for up to ten minutes. Lastly, participants completed a closing survey (survey 3) and a brief demographic survey (survey 4). Participants received $20 for completing the study and up to $10 for transportation.

### Hypotheses

This experiment sought to test several hypotheses, as detailed below:

- **H1**: Users will perceive games to have distinct attributes (as designed), and they will express a breadth of game preferences. As detailed in the Gameplay Design Section and Fig. 3, we designed each activity to have varying sensory levels, cognitive challenge, physical challenge, temporal challenge, and cooperative/competitive characteristics. Distinct game premises inspired by discussions with our game design expert are likely to satisfy users with different interests and preferences.
- **H2**: User perceptions of Baxter, including feelings of trust and opinions of the robot, will improve over the course of the study. Most participants will not have interacted with a robot in this way before, and playful interactions with a robot seem likely to lead to positive or lighthearted perceptions.
- **H3**: Younger adult participants will feel safer interacting with Baxter, affective effects of different games will vary between the younger and older age groups, and the two age groups will have different preferences in their free play game selection. Our younger participants are expected to have more experience and comfort with technology than the older adult group.
- **H4**: Gender will influence participant perceptions of Baxter and other self-reported metrics. In past related work, gender has influenced perceptions of robot sociability, positive or negative feelings toward robots, and anxieties about robots [54–57]. Any emergent differences across gender may be a useful indicator for assessing which population would most benefit from interactions with a physically interactive exercise robot.

### Analyses

Generally, our main statistical analysis tool was an 8 ×2 two-factor mixed design repeated measures analysis of variance (rANOVA) in SPSS with an *α*=0.05 significance level and game identity and age group as factors. Because of past effects of gender on affective perceptions, exercise experiences, and more, these tests also considered gender as a covariate. We calculated the effect size using eta squared, as explained further in [58].

The evaluation of **H1**, **H3**, and **H4** depended partly on rANOVA tests of responses to survey 2. The evaluation of **H2**, **H3**, and **H4** also relied at least in part on rANOVA tests on the survey 1 and 3 responses. We used rANOVA tests on raw Baxter data recordings (such as contact acceleration and motion arc length) to assess differences between subsets of our participant pool. **H2** depends on the game chosen for the final free-play period; we used a Kruskal-Wallis test with an *α*=0.05 significance level to look for differences in these preference distributions. Our analyses also relied on annotations of how many times participants tried each game and how many times users asked clarifying questions from the study video.

## Results

Before dividing the gathered data to address each hypothesis, we outline the high-level significance test results for survey responses. We then formulate our results in a way to address each individual hypothesis. When one or both main effects were significant for a particular outcome measure, post-hoc multiple comparison tests in SPSS revealed which pairs of conditions had statistically significant differences. The multiple comparisons test results appear later on, in the results corresponding to each hypothesis.

**Fig. 4:**
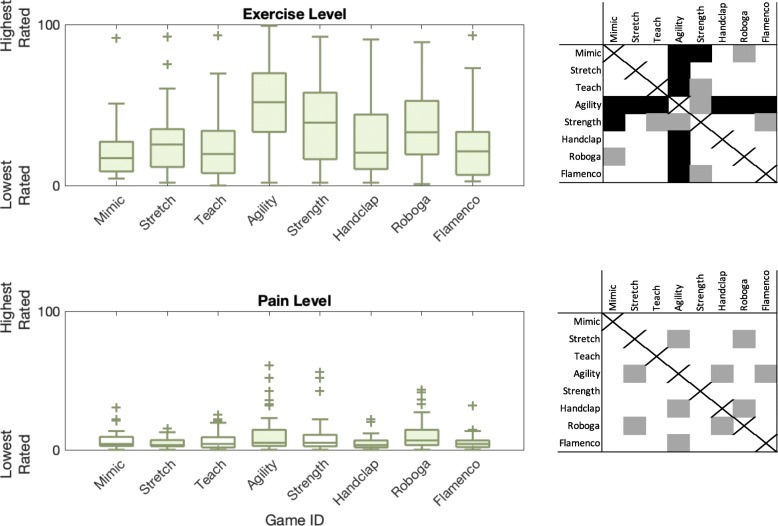
Survey responses to questions about exercise level and pain over game condition. The center box lines represent the median, and the box edges are the 25th and 75th percentiles. The whiskers show the range up to 1.5 times the interquartile range, and outliers are marked with a “+”. Filled-in box plots indicate games that were significantly differently rated compared to at least one other game. The grid next to each subplot indicates which games were rated as significantly different from others, with black indicating *p* <0.01 and gray indicating *p* = 0.01 through 0.05

At a high level, we wanted to know how game mode and participant age group affected user ratings of game exercise and pain levels in survey 2. Box plots of these raw data with indicators of significance appear in Fig. 4. Game modes had statistically significant effects on the ratings of users’ exercise level (*F*(7,266) = 10.04, *p* <0.001, *η*^2^= 0.190) and pain sensation (*F*(7,266) = 4.34, *p* <0.001, *η*^2^= 0.092). Additionally, greater participant age led to higher reported exercise levels (*F*(1,38) = 5.50, *p* =0.020, *η*^2^= 0.018; younger 28.64 ± 21.24; older 34.74 ± 25.37) and pain levels (*F*(1,38) = 22.71, *p* <0.001, *η*^2^= 0.070; younger 5.32 ± 6.98; older 10.20 ± 11.24).

**Fig. 5:**
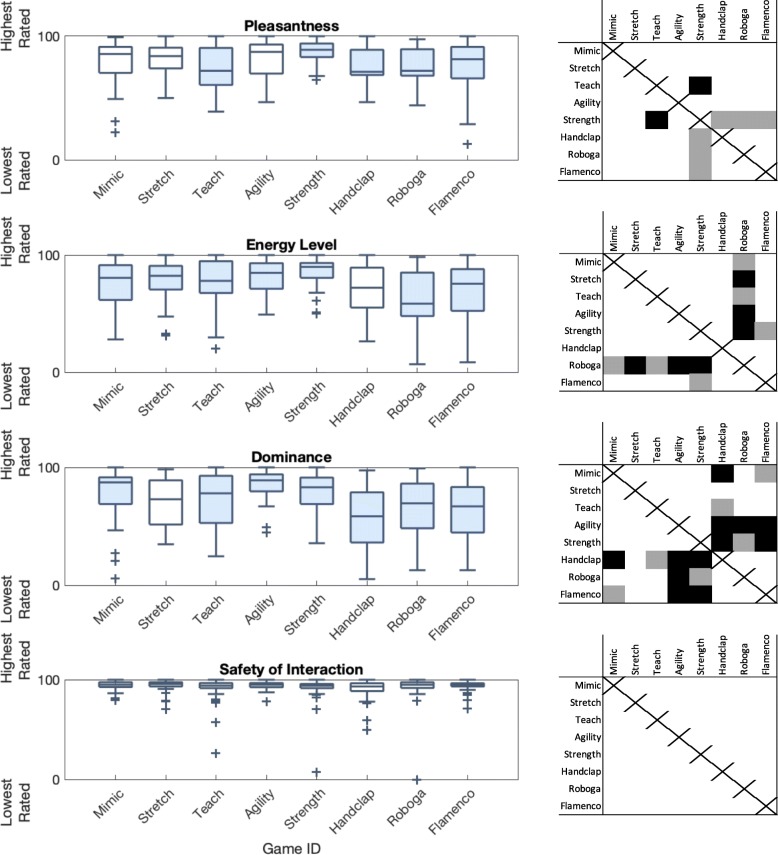
Survey responses to questions about pleasure, energy, dominance, and safety over game condition. Filled-in box plots indicate games that were significantly differently rated compared to at least one other game. The grid next to each subplot indicates which games were rated as significantly different from others, with black indicating *p* <0.01 and gray indicating *p* = 0.01 through 0.05

Additional questions in the post-exercise game survey helped us to identify how game mode and participant age group influenced user ratings of their own affect (SAM ratings) and safety feelings during interactions, as captured in survey 2. Data related to these questions appear in Fig. 5. Game mode had statistically significant effects on the ratings of user pleasantness feelings (*F*(7,266) = 3.47, *p* =0.001, *η*^2^= 0.075), energetic feelings (*F*(7,266) = 5.72, *p* <0.001, *η*^2^= 0.118), and dominance feelings (*F*(7,266) = 7.87, *p* <0.001, *η*^2^= 0.155). Older adults reported a slightly higher energy level than younger adult participants (*F*(1,38) = 4.19, *p* =0.041, *η*^2^= 0.014; younger 72.59 ± 22.54; older 77.04 ± 17.52). Game mode did not significantly influence safety ratings, and age group did not significantly influence user pleasantness, dominance, or safety ratings.

**Fig. 6:**
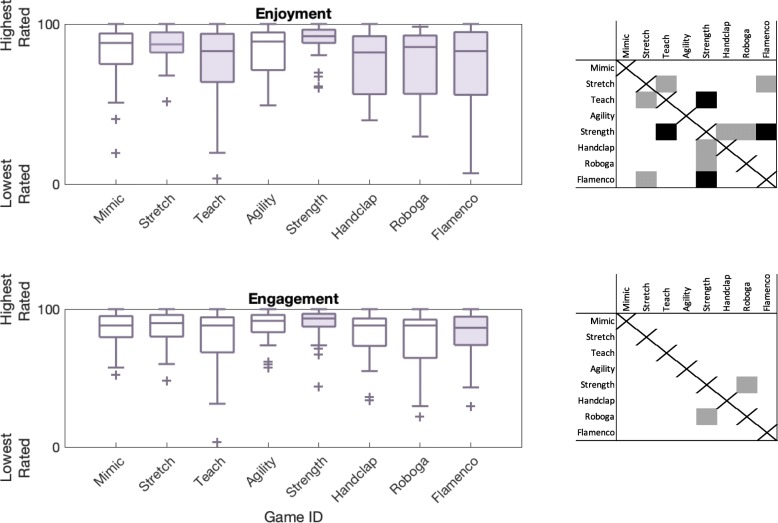
Survey responses to questions about enjoyment and engagement over game condition. Filled in box plots indicate games that were significantly differently rated compared to at least one other game. The grid next to each subplot indicates which games were rated as significantly different from others, with black indicating *p* <0.01 and gray indicating *p* = 0.01 through 0.05

We also wanted to know how game mode and participant age group influenced the user ratings of enjoyment and engagement on survey 2. Raw data and significance test results related to this topic appear in Fig. 6. Game mode had statistically significant effects on the ratings of interaction enjoyment (*F*(7,266) = 4.65, *p* <0.001, *η*^2^= 0.098) and engagement (*F*(7,266) = 3.16, *p* =0.003, *η*^2^= 0.069). Older adult users reported a slightly higher engagement level than younger participants (*F*(1,38) = 3.94, *p* = 0.048, *η*^2^= 0.013; younger 81.83 ± 18.22; older 85.28 ± 14.50). Enjoyment ratings did not differ over age group.

**Fig. 7:**
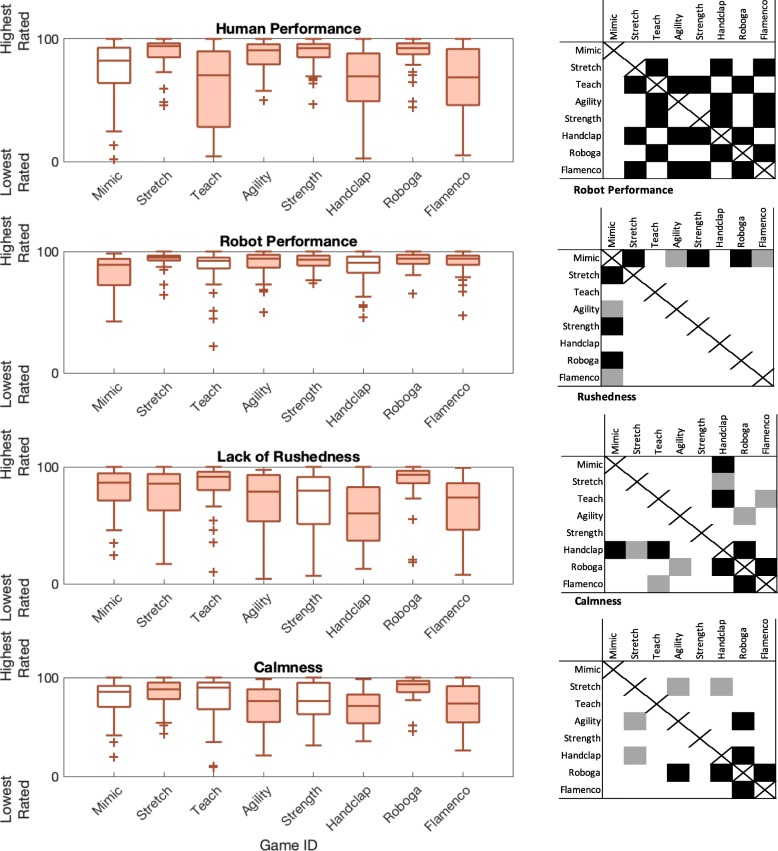
Performance- and demand-related survey responses over game condition. Filled in box plots indicate games that were significantly differently rated compared to at least one other game. The grid next to each subplot indicates which games were rated as significantly different from others, with black indicating *p* <0.01 and gray indicating *p* = 0.01 through 0.05

Lastly, we looked to identify how game mode and participant age group influenced user ratings of certain task load aspects of the exercise interactions (performance and demand questions from survey 2). There were several statistically significant trends in the responses to these survey questions, as outlined in Fig. 7. Game mode had statistically significant effects on the ratings of human performance (*F*(7,266) = 11.94, *p* <0.001, *η*^2^= 0.218), robot performance (*F*(7,266) = 4.56, *p* <0.001, *η*^2^= 0.096), rushedness during gameplay (*F*(7,266) = 6.46, *p* <0.001, *η*^2^= 0.131), and calmness during gameplay (*F*(7,266) = 5.41, *p* <0.001, *η*^2^= 0.112). Additionally, age group significantly influenced self-ratings of human performance, with older adults rating themselves lower (*F*(1,38) = 9.15, *p* = 0.003, *η*^2^= 0.030; younger 81.04 ± 20.57; older 73.01 ± 26.73). No other performance- or temporal demand-related ratings differed over age group.

### Game attributes and preferences (H1)

**Fig. 8:**
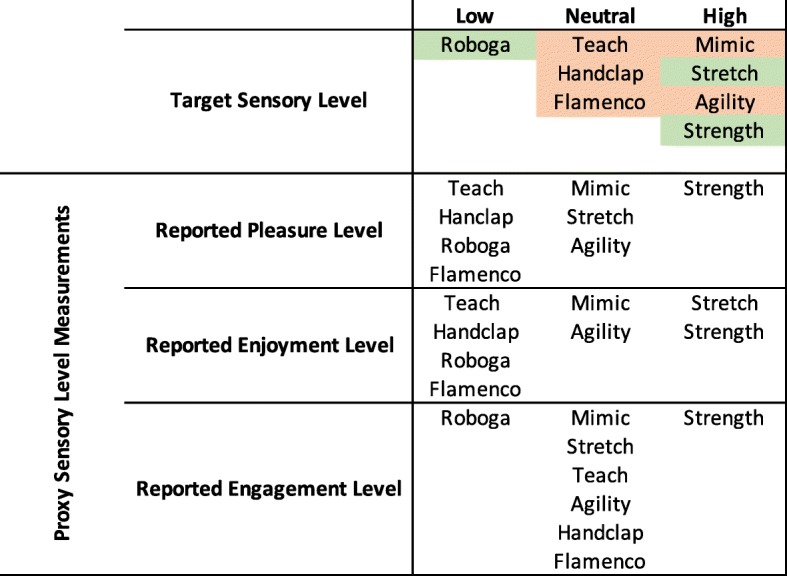
Intended engagement levels and the self-reported proxy measurements of pleasure, enjoyment, and engagement for each game. Cells in green represent games that were generally perceived as intended, while cells in orange highlight games that did not match our intended design

**Fig. 9:**
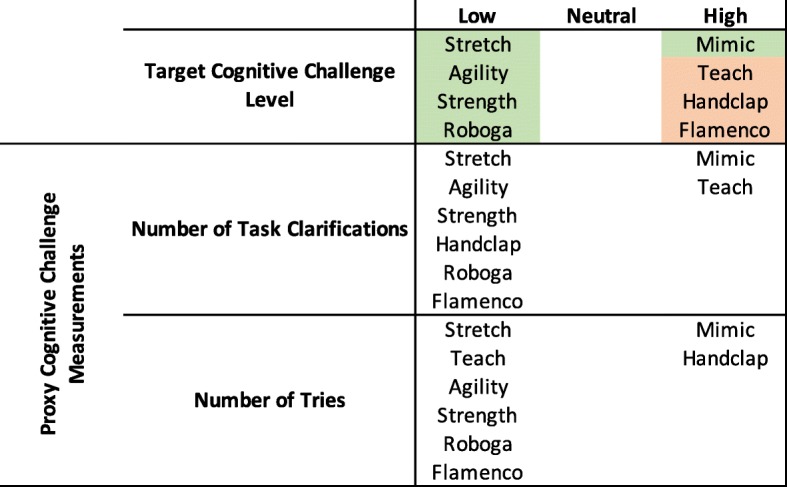
Intended cognitive challenge levels and the self-reported proxy measurements of number of task clarifications and number of tries for each game. Cells in green represent games that were generally perceived as intended, while cells in orange highlight games that did not match our intended design

**Fig. 10:**
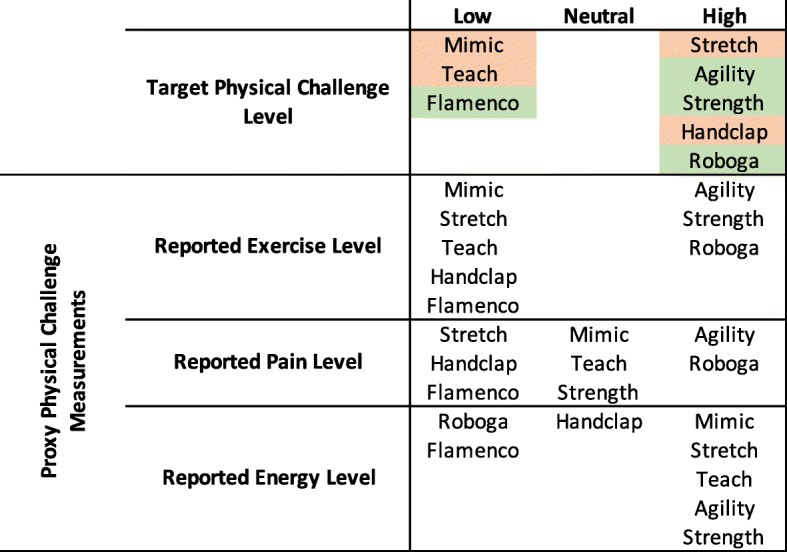
Intended physical challenge levels and the self-reported proxy measurements of exercise level, pain level, and energy level for each game. Cells in green represent games that were generally perceived as intended, while cells in orange highlight games that did not match our intended design

**Fig. 11:**
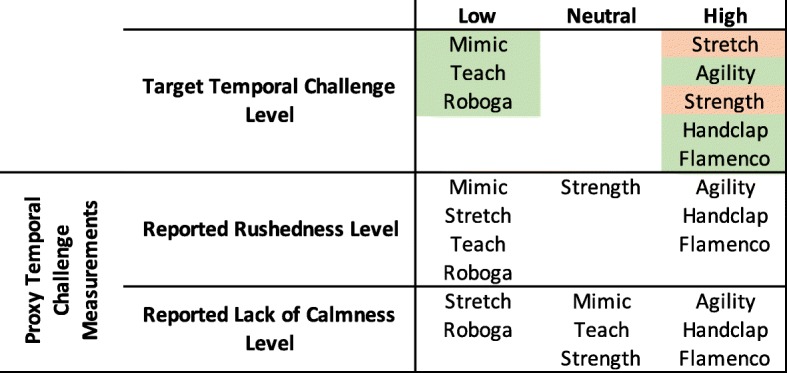
Intended temporal challenge levels and the self-reported proxy measurements of rushedness and calmness for each game. Cells in green represent games that were generally perceived as intended, while cells in orange highlight games that did not match our intended design

**Fig. 12:**
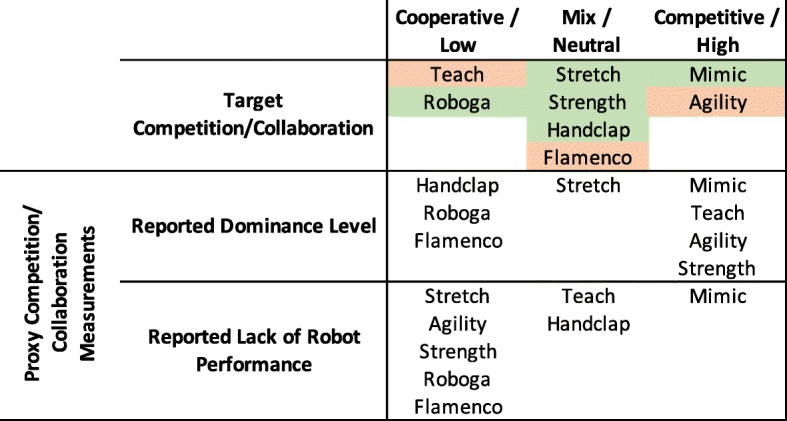
Intended competition/cooperation levels and the self-reported proxy measurements of dominance level and lack of robot performance for each game. Cells in green represent games that were generally perceived as intended, while cells in orange highlight games that did not match our intended design

As outlined previously, we designed the games to have different sensory levels, cognitive challenge, physical challenge, temporal challenge, and competitive/collaborative elements. The proxy measurements delineated in Table 2 helped us to evaluate whether or not games fit these intended qualities. Using the results of the above significance tests, we can determine whether the intended attributes matched the ones perceived by participants. For the purposes of this exploratory analysis, we consider games that fell significantly higher than at least one other game to be in the “high” category, games that fell significantly lower than at least one other game to be in the “low” category, and games that were not significantly different from any other game to be in the “neutral” category for each proxy scale. We weigh the overall “high”, “neutral”, and “low” counts for each game to determine the higher-level attributes. Figures 8, 9, 10, 11, and 12 illustrate a recap of intended attributes along with the rating tendencies for each game across appropriate proxy measurements. Games shaded in green tended to match our expectations for that particular attribute, while we did not see support for the target attribute level for games shaded in orange.

**Table 2:** Design parameters of interest and measurements that served as proxies to help us evaluate the games for each of these attributes

Engaging	Cognitively Challenging	Physically Challenging	Temporally Challenging	Competitive
Pleasure	Task clarifications	Exercise level	Rushedness	Dominance
Enjoyment	Number of tries	Energy level	Calmness	Robot performance
Engagement		Pain		

Because we expected users to rate immersive games more positively, we used self-reported pleasure, enjoyment, and engagement to assess the sensory level of each exercise game, as highlighted in Fig. 8. Three of the games trended as expected: the Stretch and Strength Games tended to be rated with higher pleasure/enjoyment/engagement, and the non-contact Roboga Game tended to be rated lower on these scales.

The metrics that we used to assess the cognitive challenge level of games were the number of task clarifications and number of tries per game. We found significant differences in both of these metrics across game (task clarifications: *F*(7,266) = 22.58, *p* <0.001, *η*^2^= 0.347, number of tries: *F*(7,266) = 17.14, *p* <0.001, *η*^2^= 0.392). Task clarifications were significantly more common in the Teach Game (1.50 ± 1.24) than in any other game. The Mimic Game (0.56 ± 0.99) also elicited significantly more clarifications than the Stretch, Strength, and Roboga Games (all ≤0.10 ± 0.31). If participants lost the exercise games because of a misunderstanding about the gameplay rules, the research assistant allowed them to try the game again. Participants required significantly more tries to master the Mimic Game (1.72 ± 0.94) than any other game. The Handclap Game (1.32 ± 0.47) led to more tries than any other game except the Mimic and Teach Games (all ≤ 1.02 ± 0.16). All eight participants who required three or more tries on the Mimic Game were in the older adult group, and three of them exhibited signs of mild cognitive impairment on the opening MoCA assessment. Overall, the Mimic, Stretch, Agility, Strength, and Roboga Games trended as expected. The Mimic Game was more cognitively challenging, and the Stretch, Agility, Strength, and Roboga Games were less challenging in this area.

Self-reported exercise level, pain, and energy level served as proxies for the physical challenge of exercise games, as detailed in Fig. 10. Our understanding of the games was mixed in this category; four of the games trended as expected, but the others were perceived in a mixed or opposite way. The Agility, Strength, and Roboga Games were generally correctly predicted as higher exercise level/pain level/energy level, and the Flamenco Game was correctly predicted as lower in these categories.

We used self-reported rushedness and calmness levels as proxies for temporal challenge, as illustrated in Fig. 11. Six of the games trended as expected: the Agility, Handclap, and Flamenco Games tended to be rated with higher rushedness/lower calmness, and the Mimic, Teach, and Roboga Games tended to be rated lower on these scales.

We assessed the competitive or collaborative nature of different games with the help of self-reported dominance and the inverse of robot performance ratings, as illustrated in Fig. 12. Five of the games trended as expected: the Mimic Game tended to be rated as more competitive, the Stretch, Strength, and Handclap Game were rated in a mixed or neutral way, and the Roboga Game was rated as lower/more collaborative.

**Fig. 13:**
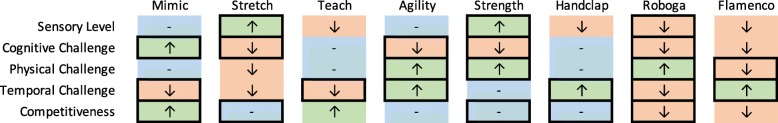
Actual perceived game characteristics of each human-robot exercise game. Outlined boxes indicate attributes perceived as expected

Figure 13 summarizes the game characteristics, outlining boxes that were perceived by participants as we expected. Overall, more than half of the game attributes were perceived as intended. The Mimic, Stretch, Agility, Strength, and Roboga Games resulted in perceptions most similar to our expectations (majority of correct attributes), so we view these games as closest to our designed intentions.

**Fig. 14:**
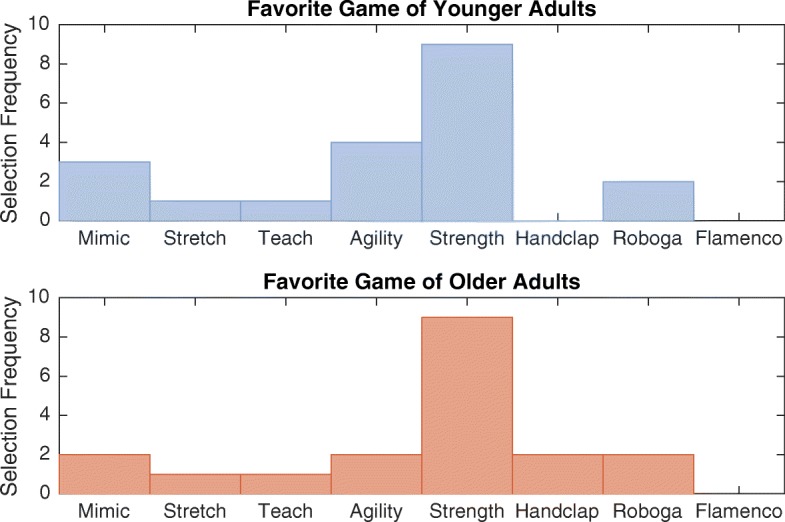
Favorite games of the two participant age groups

All users were able to identify a favorite game that they wanted to play again, and every participant interacted with Baxter in this free-play game condition for at least as long as the sample game interactions. Several people played many repetitions of their chosen game, and some opted to increase the difficulty level over their free-play game experiences. As illustrated in Fig. 14, the Strength Game was the most popular choice, but aside from the Flamenco Game, every game was chosen as a favorite by at least two users. A Kruskal-Wallis test revealed no significant difference between the game selections of younger and older adults (*p* = 0.365).

### Changing perceptions of Baxter (H2)

**Fig. 15:**
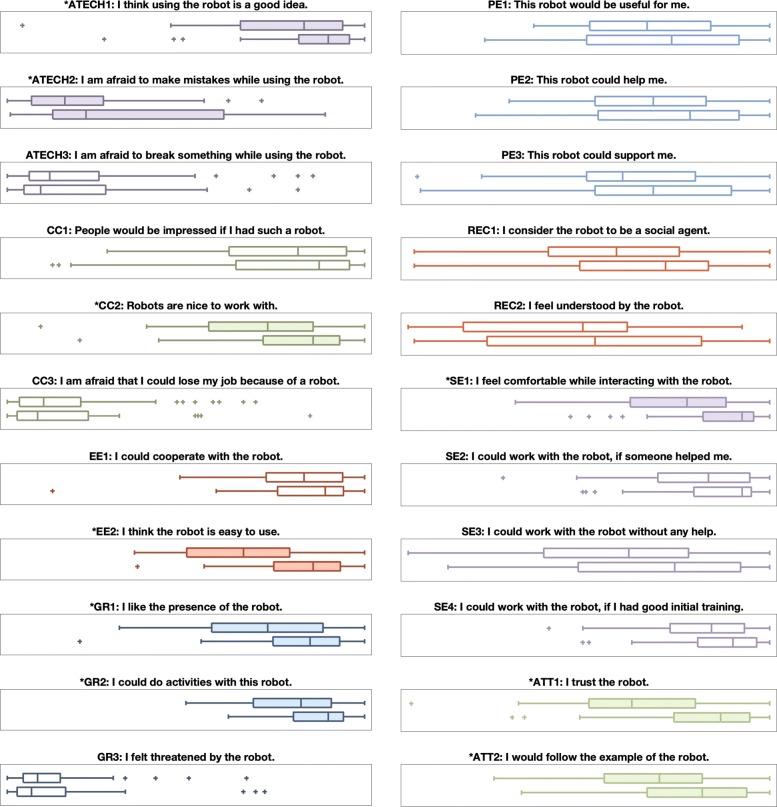
Differences in responses to the UTAUT-inspired robot perception survey. In each plot, the top box plot represents the pre-experiment responses, and the bottom box plot represents the post-experiment responses. Filled in box plots with starred titles indicate significant differences. Clusters of plots with matching colors and letter codings represent survey question groupings

We gathered two sets of robot perception survey responses, one before and one after the experiment; these results are shown in Fig. 15. Because we gathered these data from two different participant age groups, our main analysis tool for evaluating differences in the robot perception survey was a 2 ×2 two-factor mixed design rANOVA performed in SPSS with an *α*=0.05 significance level. We additionally calculated effect sizes using eta squared. The before/after results are discussed here, and the age-related results of this analysis appear in the following section.

Based on this analysis, we discovered that over the course of the experiment, users became more positive about the idea of using the robot (*F*(1,38) = 4.38, *p* = 0.045, *η*^2^= 0.054) but more afraid to make mistakes while playing with Baxter (*F*(1,38) = 5.40, *p* = 0.023, *η*^2^= 0.069). Participants also came to think that the robot was nicer to work with (*F*(1,38) = 5.28, *p* = 0.024, *η*^2^= 0.068) and easier to use (*F*(1,38) = 19.81, *p* <0.001, *η*^2^= 0.213) after the experiment. Users further reported liking the presence of the robot more (*F*(1,38) = 7.63, *p* = 0.007, *η*^2^= 0.095) and being more able to imagine doing activities with the robot (*F*(1,38) = 7.41, *p* = 0.008, *η*^2^= 0.092). Ratings of comfort interacting with the robot also increased (*F*(1,38) = 11.75, *p* = 0.001, *η*^2^= 0.139). Lastly, respondents were more trusting of Baxter (*F*(1,38) = 16.76, *p* <0.001, *η*^2^= 0.187) and more willing to follow Baxter’s example (*F*(1,38) = 11.83, *p* = 0.001, *η*^2^= 0.139) after the experiment. It is important to note that these findings were true for both younger and older adults.

### Age differences (H3)

**Fig. 16:**
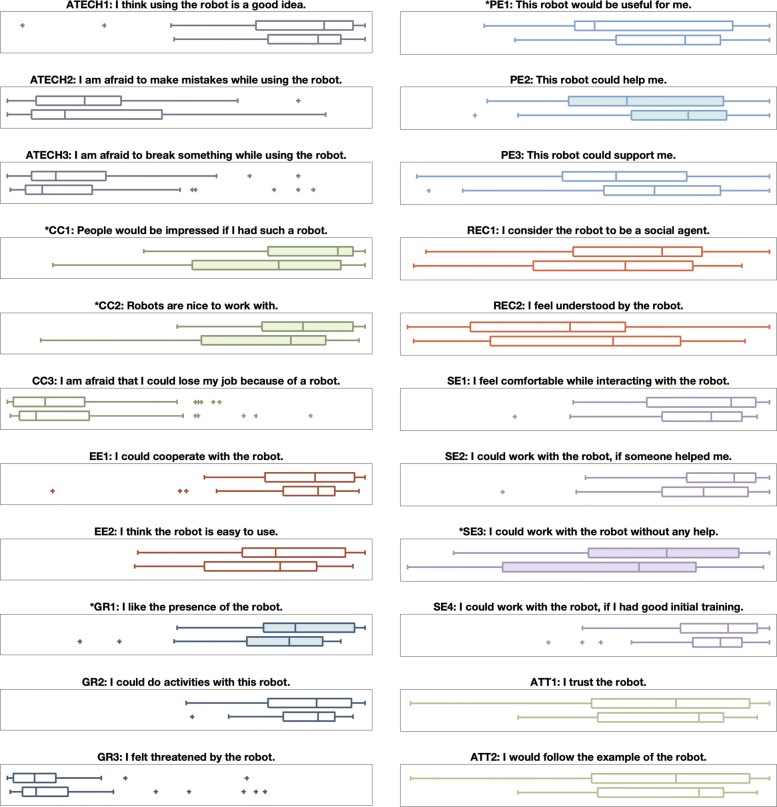
Differences in responses to the UTAUT-inspired robot perception survey. In each plot, the top box plot represents the younger adult responses, and the bottom box plot represents the older adult responses. Clusters of plots with matching colors and letter codings represent survey question groupings

Certain differences were present in the younger vs. older adult perceptions of Baxter, as shown in Fig. 16. Younger adults agreed more with the statement that others would be impressed if they had a robot like Baxter (*F*(1,38) = 6.45, *p* = 0.013, *η*^2^= 0.081), and the younger group also thought robots are nicer to work with (*F*(1,38) = 4.41, *p* = 0.039, *η*^2^= 0.057). The younger group liked the presence of Baxter more (*F*(1,38) = 3.71, *p* = 0.042, *η*^2^= 0.056). In contrast, the older adults agreed more strongly that Baxter could help them (*F*(1,38) = 4.36, *p* = 0.040, *η*^2^= 0.056). Lastly, younger adults felt more confident about using Baxter without any help (*F*(1,38) = 4.18, *p* = 0.044, *η*^2^= 0.054).

Age-related differences also appeared in post-game surveys and data recordings from younger vs. older adult study participants. As reported previously in the main effects, older adult participants felt like they were exercising harder and felt more pain during the experiment. Older adults also rated themselves as more energetic than younger adult participants, and the older participant group reported being more engaged than younger participants. Additionally, age group significantly influenced self-ratings of human performance; older adults generally rated themselves as performing worse.

There were no significant differences between the participant-wise mean accelerations at hand impact for younger and older adult participants (lowest *p* = 0.140). Differences did appear in arc length moved during the Teach Game; older adults moved both the right and left robot arms through higher arc length distances during this activity (left: *F*(1,39) = 5.12, *p* = 0.030, *η*^2^= 0.122; right: *F*(1,39) = 4.11, *p* = 0.050, *η*^2^= 0.100), which may indicate that they were more motivated to explore the space and compose a song during this game in particular.

### Gender effects (H4)

Throughout many of the tests described above, including gender as a covariate revealed differences between the responses of participating men and women. Certain differences arose in the participants’ overall opinions of Baxter; women agreed significantly more with all of the following statements:

- I think using the robot is a good idea (*F*(1,38) = 11.66, *p* = 0.001, *η*^2^= 0.138)
- People would be impressed if I had such a robot (*F*(1,38) = 8.46, *p* = 0.005, *η*^2^= 0.104)
- Robots are nice to work with (*F*(1,38) = 4.30, *p* = 0.042, *η*^2^= 0.056)
- I could cooperate with the robot (*F*(1,38) = 5.72, *p* = 0.019, *η*^2^= 0.073)
- I like the presence of the robot (*F*(1,38) = 7.66, *p* = 0.007, *η*^2^= 0.095)
- I consider the robot to be a social agent (*F*(1,38) = 4.58, *p* = 0.036, *η*^2^= 0.059)
- I feel understood by the robot (*F*(1,38) = 8.08, *p* = 0.006, *η*^2^= 0.100)
- I trust the robot (*F*(1,38) = 14.09, *p* <0.001, *η*^2^= 0.162)
- I would follow the example of the robot (*F*(1,38) = 24.34, *p* <0.001, *η*^2^= 0.250)

Self-reported metrics also differed occasionally between male and female participants. In these survey responses, female participants rated all of the following significantly higher than men:

- Pain (*F*(7,266) = 8.42, *p* = 0.004, *η*^2^= 0.027)
- Robot performance (*F*(7,266) = 19.33, *p* <0.001, *η*^2^= 0.061)
- Lack of rushedness (*F*(7,266) = 12.30, *p* = 0.001, *η*^2^= 0.039)
- Calmness (*F*(7,266) = 17.04, *p* <0.001, *η*^2^= 0.054)

These effects held true for both younger and older participants.

## Discussion

The study results enable us to evaluate our hypotheses about the human-robot exercise games, reflect on strengths and limitations of this work, and make recommendations about how this exploratory study can inform future work.

### Hypothesis testing

Five of the eight exercise games mostly matched the expected attributes referenced in **H1**: the Mimic, Stretch, Agility, Strength, and Roboga Games. Three games did not fulfill our intentions for how participants would feel during gameplay. The Teach Game fit only one of our expectations, and the Handclap and Flamenco Games met only two. The most popular activity among participants was the Strength Game, perhaps because of its use of energetic and recognizable music. Even so, participant preferences spanned almost all other games. We consider these results as preliminary evidence that we successfully designed an array of games suitable to users with different interests, fitness goals, physical abilities, and cognitive needs.

The experience of interacting with Baxter influenced user perception of the robot, as anticipated in **H2**. The strongest effects were increases in user trust of the robot and feelings that the robot is easy to use (both *η*^2^ > 0.18). Perception of using the robot, how the robot is to work with, the presence of the robot, doing activities with the robot, comfort with the robot, and willingness to follow the robot’s example all increased significantly as well. These findings align well with past qualitative observations from [59].

Although there were several differences between younger and older adult participant responses to the games, almost none of the differences postulated in **H3** were upheld. Overall, younger participants did give higher ratings of confidence about using Baxter, being around Baxter, and Baxter’s impressiveness than older participants, but the older adults thought Baxter would be more able to help them. Older adult participants seemed to get more out of the exercise interactions, as intended; they felt more energetic and engaged during exercise, and they also reported higher exercise and pain levels than younger adults. The cause of most reported pain was muscle burn from exercise, but we recommend caregiver supervision to monitor pain levels. Older adults also rated their own performance as worse, which may be beneficial in this work; if we aim to create games that are challenging but possible, this balance may ultimately lead to more satisfaction and feelings of self-efficacy [60].

As anticipated in **H4**, female participants perceived the robot differently than men on several pre- and post-study survey scales. Overall, female users’ opinion of Baxter was higher, and they also experienced more pain (usually muscle burn- or contact comfort-related) during the exercise games. These results, in combination with the positive feedback from older female users of the system, may indicate that women are better target users for this type of exercise system, both in terms of acceptance and potential exercise gains. The all-female composition of our research team might also be partially responsible for these gender differences.

### Major strengths and limitations

The results of this experiment showed us how younger and older adults respond to exercise games with a robot. Participants felt safe physically interacting with Baxter and playing the exercise games. Most games were perceived approximately as expected, fulfilling a majority of the design attributes that we aimed to deliver in each activity. This result gives us confidence in the ability of researchers to use similar strategies in the future design of more targeted interventions.

User preferences were split across activities, and each game seemed to possess characteristic advantages as follows:

- **Mimic Game**: Although participants often needed multiple tries to understand this game, it was clearly understood to be a competitive game. Participants’ perceptions of the activity were mostly aligned with our intended design criteria.
- **Stretch Game**: This game achieved most of our intended design characteristics. It seemed to be one of the most attainable and relaxing games for users.
- **Teach Game**: This game was not interpreted as we intended, but this activity prompted many questions from participants. Some users were uninterested in the musical premise, but others became determined and thoroughly explored the robot’s workspace during their trial.
- **Agility Game**: Participants worked harder than expected during this game, leading to high physical and cognitive challenge. A frequent favorite of participants, this game fulfilled most of our design attributes.
- **Strength Game**: This game achieved most of our intended design characteristics and was the most frequent favorite game choice of participants. Participants were generally very positive about this activity, and the Strength Game led to higher reports of exercise than several other games.
- **Handclap Game**: Participants experienced some of the highest cognitive challenge levels when playing this game, because of both spatial awareness and memory demands.
- **Roboga Game**: This activity met most of our design expectations. The game was simple to grasp (no participants needed instruction clarifications or additional chances to try the game).
- **Flamenco Game**: Although no participants chose this activity for the free play period, this game led to a playful interaction, and many individuals reported enjoying the game in their spoken and written game commentary.

Only *socially and physically interactive* games **fell in the highest ranges for pleasantness, enjoyment, engagement, cognitive challenge, energy level, and competitiveness**.

Users typically finished or won all the games, which emphasizes the readability and comprehensibility of the activities. This result was ideal because we wanted to verify that people can succeed in the games before testing more challenging or vigorous game modes. Doing this type of activity with a robot before a higher-stakes team interaction may help the pair to build rapport and trust; users’ opinions of and trust in the robot improved over the course of the study.

Certain limitations arose from the study design. Although the user population was diverse, users were not uniformly representative of the target population for this research. Collecting data from a larger and more diverse sample could increase the strength of our results. Likewise, the lab setting of the experiment and the short duration of each interaction did not perfectly match our intended use case. To ensure broader applicability, it would be ideal to run a similar experiment in an assisted living facility. Additionally, the within-subjects nature of the experiment may have exaggerated differences between game conditions due to demand characteristics.

Users reported a growing fear of making mistakes when interacting with Baxter. This change is likely a byproduct of the ability to lose the exercise games by making mistakes, but we will monitor for this same concern in future studies and seek to understand why this change occurs. The implementation of the exercise games stands to be improved before future deployments; the operation of the robot was nearly, but not yet fully, autonomous. Another drawback was that one user broke Baxter’s wrist motor coupler (W2 joint). In similar future exercise studies, we recommend developing a protocol to service the robot between study sessions as needed and supplying cautionary feedback if participants hit the robot with excessive force.

## Conclusions

Overall, the positive results of this study show that social-physical exercise with a human-sized humanoid robot has the potential to encourage physical and cognitive exercise by older adults. Both younger and older participants felt that the robot was safe and were willing to play the games. Games involving both social and physical interaction were rated as most pleasant, enjoyable, engaging, cognitively challenging, and energetic. The games also successfully achieved different physical, cognitive, and temporal challenge levels. The social aspects of interactions were especially well received by female users. Older users’ higher energy level, exercise, and pain ratings show that these games are more relevant to the exercise of older individuals.

Researchers working on related topics can learn from the iterative game design approach that we followed, the open-source resources we provide, and the scientific results from our research. Our work with clinicians and other experts during exercise game design helped us to propose safe, entertaining, and beneficial interactions with various built-in song/pattern modes and difficulty levels to preserve interest in the robot over multiple interactions. The code and instructions needed to run the exercise games from our study are publicly available at [53]. Our game-specific findings relate most closely to the specific activities investigated in this work, but other results (e.g., experience differences across age group and gender, implications of social-physical interactions generally) can guide assistive robotics work more broadly.

In our own future steps, we see potential to adjust games or select a subset of games to accomplish specific physical therapy goals; for example, we can emphasize physical, cognitive, or temporal challenge. Our future investigations of social-physical robots as exercise partners will help us understand how to use these agents to support older adults and other individuals with exercise needs.

## Appendix A: Exercise Game Descriptions

Here, we outline additional information about the games used in our study. We further describe the logical flow of each game in figures and videos included as supplementary material with this article. (Additional files 1, 2, 3, 4, 5, 6, 7, 8, 9, 10, 11, 12, 13, 14, 15, 16, and 17)

The **Mimic Game** was designed to make users hold up their arms and contact Baxter’s end-effectors. In this game, the user gradually teaches Baxter a long pattern of left-, right-, and/or both-handed impacts which Baxter has to repeat during each round of the activity. The participant can win the game by demonstrating a sequence of hand impacts that is long enough to “confuse” the robot. Specifically, based on the participant cognitive wellness level assessed by our opening MoCA evaluation, we set the number of motions needed to win at 3-6. To discourage the case where participants could repeat the same motion again and again to win, we counted motions repeated in direct sequence as only half of a motion. The human user loses if they make a mistake when repeating their own hand impact pattern. This game involves a cognitive dimension that challenges the user to remember a pattern. We believed this game would involve a high sensory level because of the energetic hand contacts in the interaction and intermittent percussive sounds associated with each gameplay move. The Mimic Game was designed to be a cognitively challenging and competitive experience with low physical and temporal demand.

The **Stretch Game** leverages Baxter’s sizable workspace to encourage the user to make large arm motions. In this interaction, Baxter strikes a series of poses, cuing the user to mimic its pose and simultaneously hit both of its end-effectors within a fixed time after reaching each new pose. At the end of the game, Baxter plays a series of chords for all of the presented poses, with dissonant chords to represent any contacts that the user missed. People engaging with the robot must use their spatial awareness to reposition themselves and their arms as needed throughout the game. We thought this game would involve a high sensory level because of the energetic hand contacts involved in the gameplay and intermittent chords associated with each gameplay pose. The Stretch Game was designed to be a physically demanding, temporally challenging, and part-competitive/part-collaborative experience with low cognitive demand.

The **Teach Game** challenges users to support Baxter’s arms while moving them around the robot’s workspace to create a musical composition. The user can play notes by twisting just one of Baxter’s wrists; arm locations map to notes. Chords are recorded to the composition when the user twists both of Baxter’s wrists simultaneously. Once the user is done composing, Baxter plays back the recorded sequence of notes with the associated motions. If the user intends to create a particular composition, the game requires cognitive abstraction skills (to understand how the robot’s pose relates to a musical note) and attention (to be able to explore the workspace and select notes before losing track of current and past notes). We thought this activity would involve a moderate sensory level because the participant is more passively contacting Baxter (holding the robot’s arms) and the game involves intermittent chord sounds. The Teach Game was designed to be a cognitively demanding and collaborative experience with low physical and temporal challenge.

The **Agility Game** was designed to encourage users to hold up their arms and rapidly contact Baxter. In this interaction, the user attempts to “wake” a sleeping Baxter by repeatedly hitting its end-effectors. This activity requires fast, but not necessarily forceful, hand contact. We believed this game would involve a high sensory level because of the energetic hand contacts and the intermittent sound effects involved in the interaction. The Agility Game was designed to be a physically challenging, temporally challenging, and competitive experience with low cognitive demand.

The **Strength Game** encourages users to hit Baxter somewhat forcefully while going through a boxing training-like interaction set to energetic music. Baxter strikes a sequence of poses and prompts the user to contact its end-effectors with a punch to each boxing pad in every subsequent pose. This game requires attention (to perceive the cues indicating that Baxter is ready for contact). We thought this game would involve a high sensory level because of the energetic hand contacts and music involved in the gameplay. The Strength Game was designed to be a physically challenging, temporally challenging, and part-collaborative/part-competitive experience with low cognitive demand.

The **Handclap Game** was designed to make users hold up their arms and contact Baxter’s end-effectors. This interaction is similar to a children’s hand-clapping game, such as “Pat-a-Cake” or “Miss Mary Mack.” Baxter demonstrates a series of hand-clapping motions, and the user joins in the clapping game by physically contacting the robot’s hands. The same hand-clapping game repeats with one new appended motion in each repetition. Users are challenged in visuospatial cognition as they interpret and reciprocate robot movements. We believed this game would involve a medium sensory level because although the interaction involves energetic hand contacts, there is no accompanying sound. The Handclap Game was designed to be a cognitively challenging, physically demanding, temporally challenging, and part-competitive/part-collaborative experience.

The **Roboga Game** is similar to related work in [7] and does not involve physical human-robot contact. Users are challenged by the need to hold up their own arms’ weight. Baxter strikes a stretching pose, the user matches the pose, and both parties hold the pose for several seconds. The Roboga Game has a set length, and the game concludes when the robot completes its sequence of movements. There is no feedback or automated user monitoring, although we found that users attempted to mimic the robot in all cases but one (during an inquisitive free-play trial). The poses are concatenated to create stretching routines similar to those found in physical therapy exercises for shoulder and bicep tendon injuries, and potentially beneficial for general strength and flexibility. Because of the lack of physical contact and sound during this activity, we expected this game to have a lower sensory level. Overall, the Roboga Game was designed to be a physically demanding and collaborative experience that is less cognitively and temporally challenging.

The **Flamenco Game** challenges users to exercise by carrying out different dance moves, none of which involve physical human-robot contact. Baxter demonstrates a sequence of simple dance moves along with music, nods to the participant, and then waits for the human user to try the same dance along with a music replay. The researcher manually started each sequence of robot dance moves after the music replay from the previous sequence concluded. There was no user feedback during this game, but we found that participants always attempted the dance replay. Users are challenged in visuospatial cognition as they interpret and reciprocate robot movements. Since this game involved no physical contact but did involve energetic music, we expected this game to involve a medium sensory level. The Flamenco Game was designed to be a cognitively challenging, temporally challenging, and part-competitive/part-collaborative experience that is less physically demanding.

## Supplementary information

**Additional file 1** Gameplay flow of the Mimic Game.

**Additional file 3** Gameplay flow of the Stretch Game.

**Additional file 5** Gameplay flow of the Teach Game.

**Additional file 7** Gameplay flow of the Agility Game.

**Additional file 9** Gameplay flow of the Strength Game.

**Additional file 11** Gameplay flow of the Handclap Game.

**Additional file 13** Gameplay flow of the Roboga Game.

**Additional file 15** Gameplay flow of the Flamenco Game.

**Additional file 18** Opening survey (survey 1) completed by study participants.

**Additional file 19** Post-game survey (survey 2) completed by study participants.

**Additional file 20** Closing survey (survey 3) completed by study participants.

**Additional file 21** Demographic survey (survey 4) completed by study participants.

## Data Availability

The full datasets generated and analyzed during the current study are not publicly available based on the terms of our IRB protocol, but they are available to individuals upon their addition to the protocol. This modification may be accomplished upon reasonable request with the approval of the Penn IRB. We believe that the exercise games themselves are a key contribution of this work, so we have attempted to provide the information needed for interested parties to reproduce and build on these games. A video explaining each of the exercise games is available here: https://www.youtube.com/watch?v=5zlaqlJJpts&feature=youtu.be. Interested parties can download the source code for our games here: https://github.com/shareresearchteam/baxter-exercise-games. In this repository, we include details on how to launch the exercise games in the same way that we did in our study.

## Abbreviations

BDI: Beck’s depression inventory
BnB: Box and blocks
MoCA: Montreal cognitive assessment
NSF: National science foundation
Penn: University of Pennsylvania
pHRI: physical human-robot interaction
rANOVA: repeated measures analysis of variance
SAM: Self-assessment manikin
TLX: Task load index
UTAUT: Unified theory of acceptance and use of technology
