# Supplementary figures and images for: Exercising with Baxter: preliminary support for assistive social-physical human-robot interaction
Source: J Neuroeng Rehabil. 2020 Feb 17;17:19. doi: 10.1186/s12984-020-0642-5 (PMC7027056; doi:10.1186/s12984-020-0642-5)

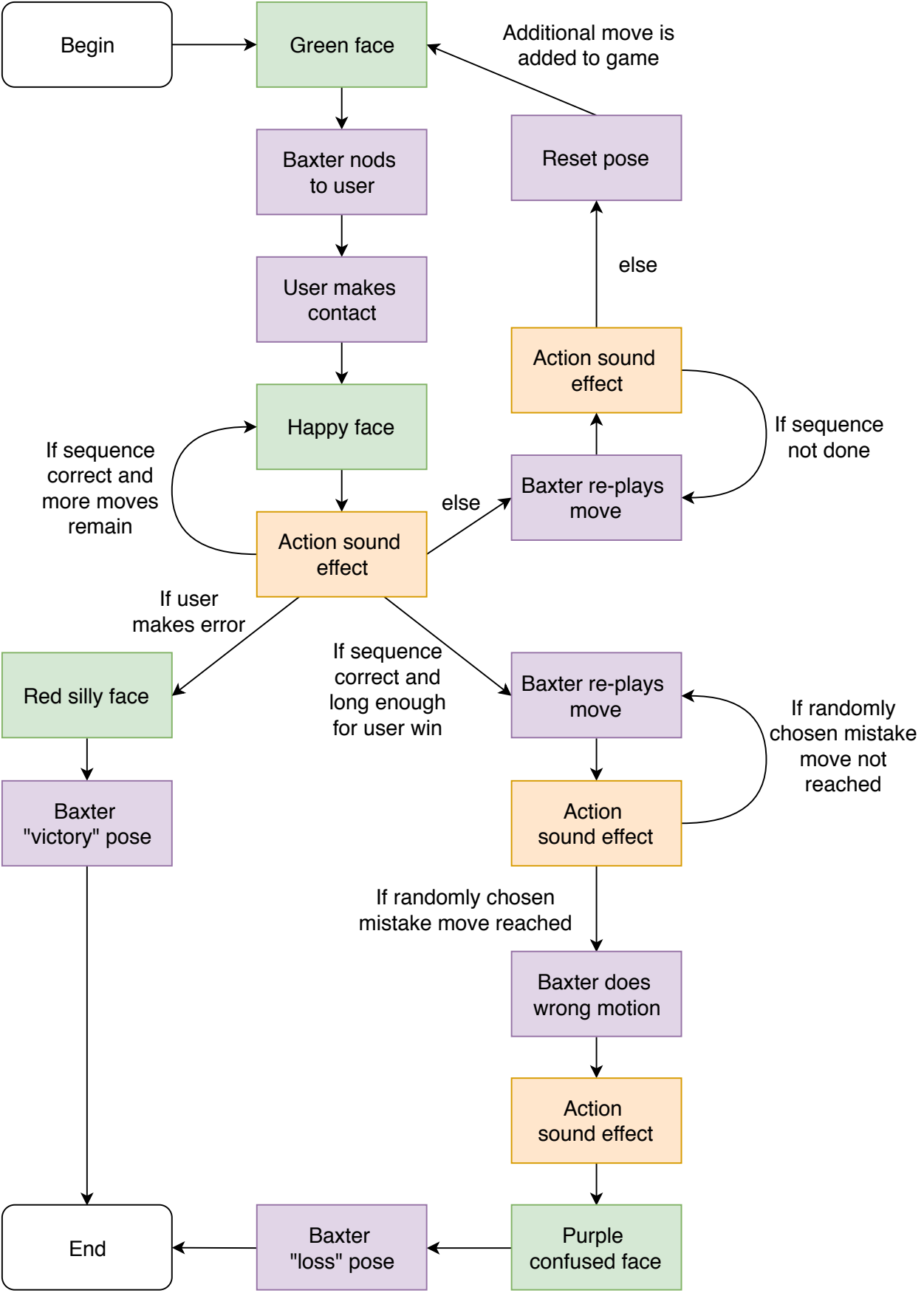

Supplement: Supplementary file 10 — Additional file 1 Gameplay flow of the Mimic Game. [file 12984_2020_642_MOESM1_ESM.pdf]

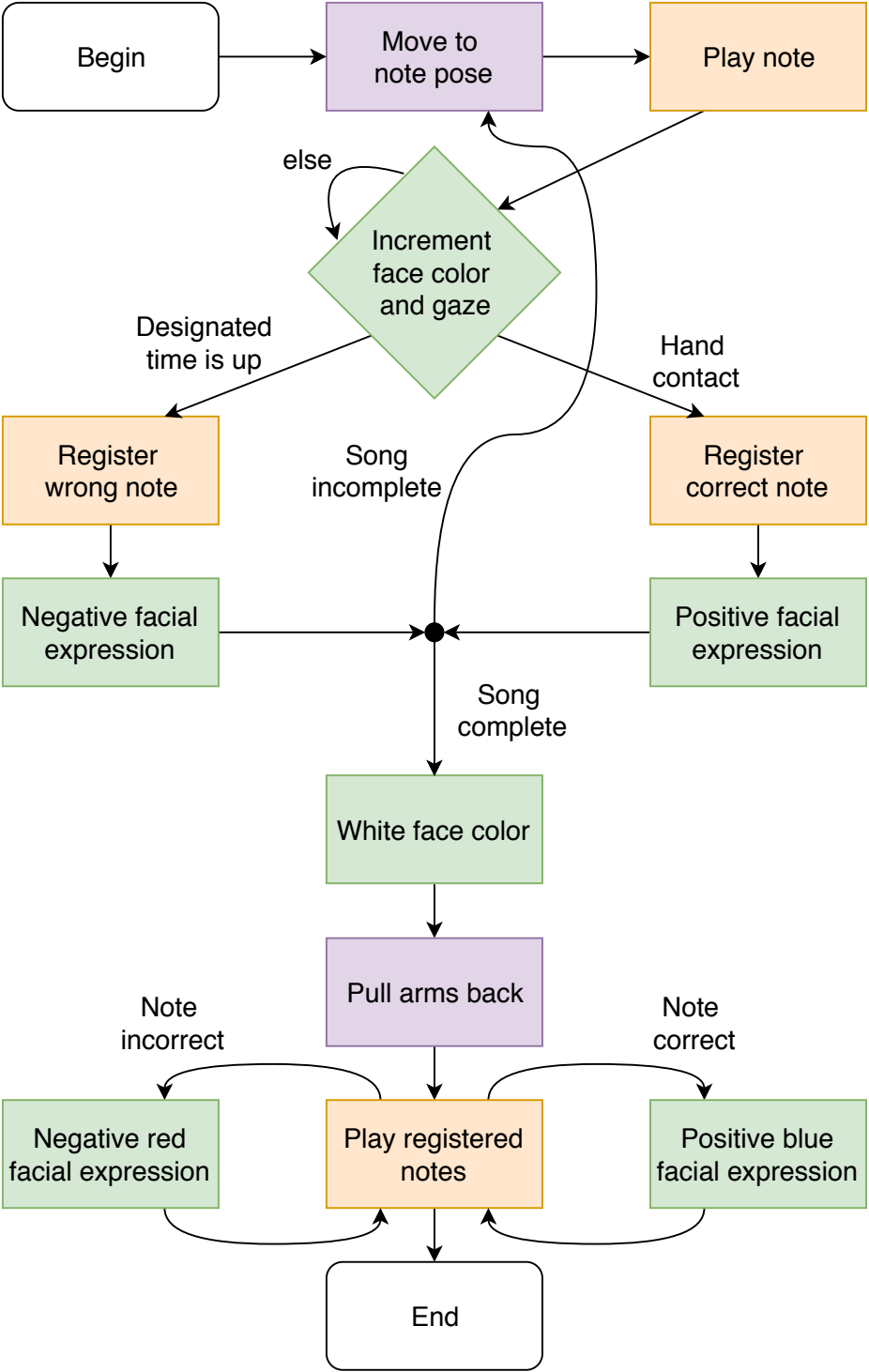

Supplement: Supplementary file 11 — Additional file 3 Gameplay flow of the Stretch Game. [file 12984_2020_642_MOESM3_ESM.pdf]

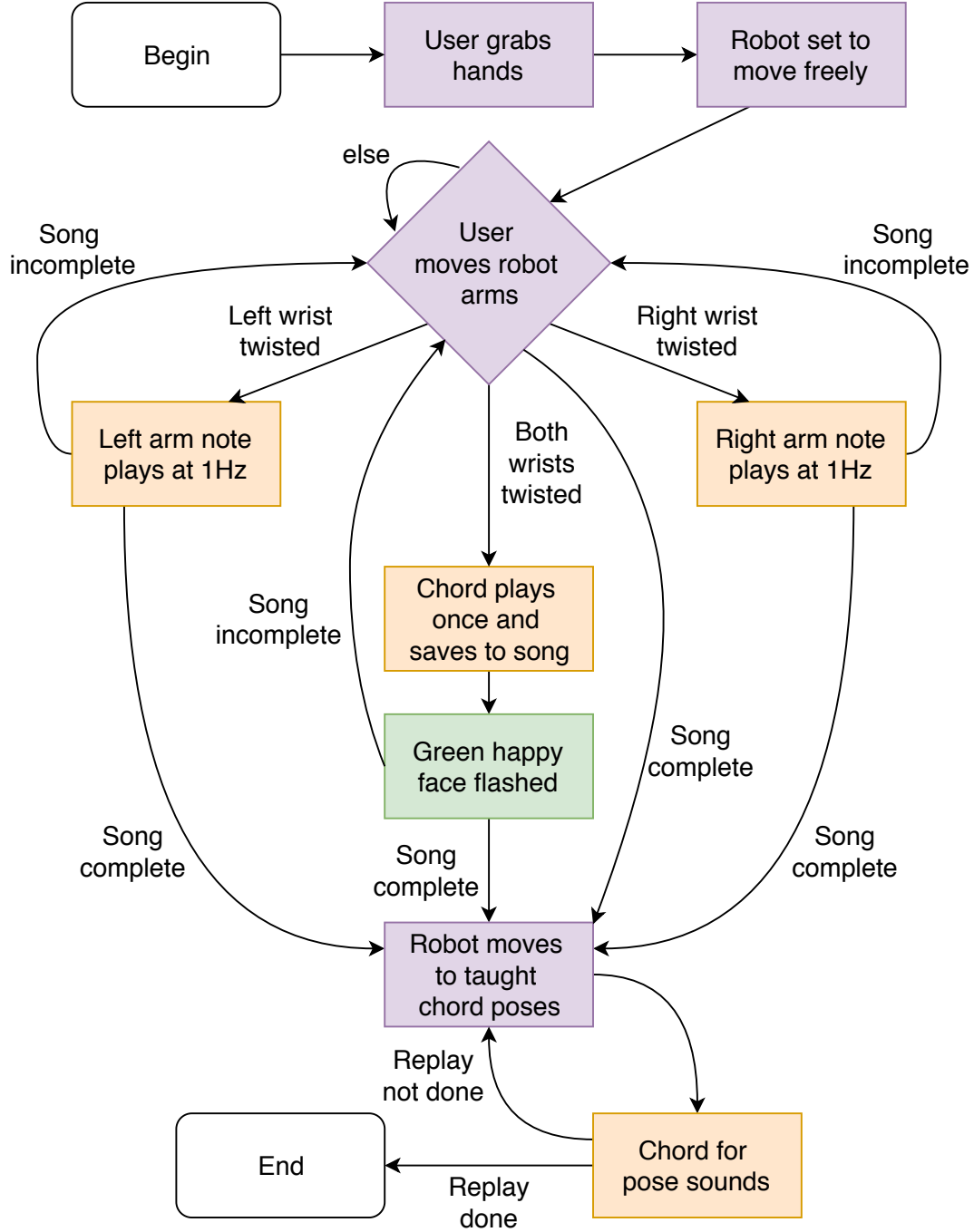

Supplement: Supplementary file 12 — Additional file 5 Gameplay flow of the Teach Game. [file 12984_2020_642_MOESM5_ESM.pdf]

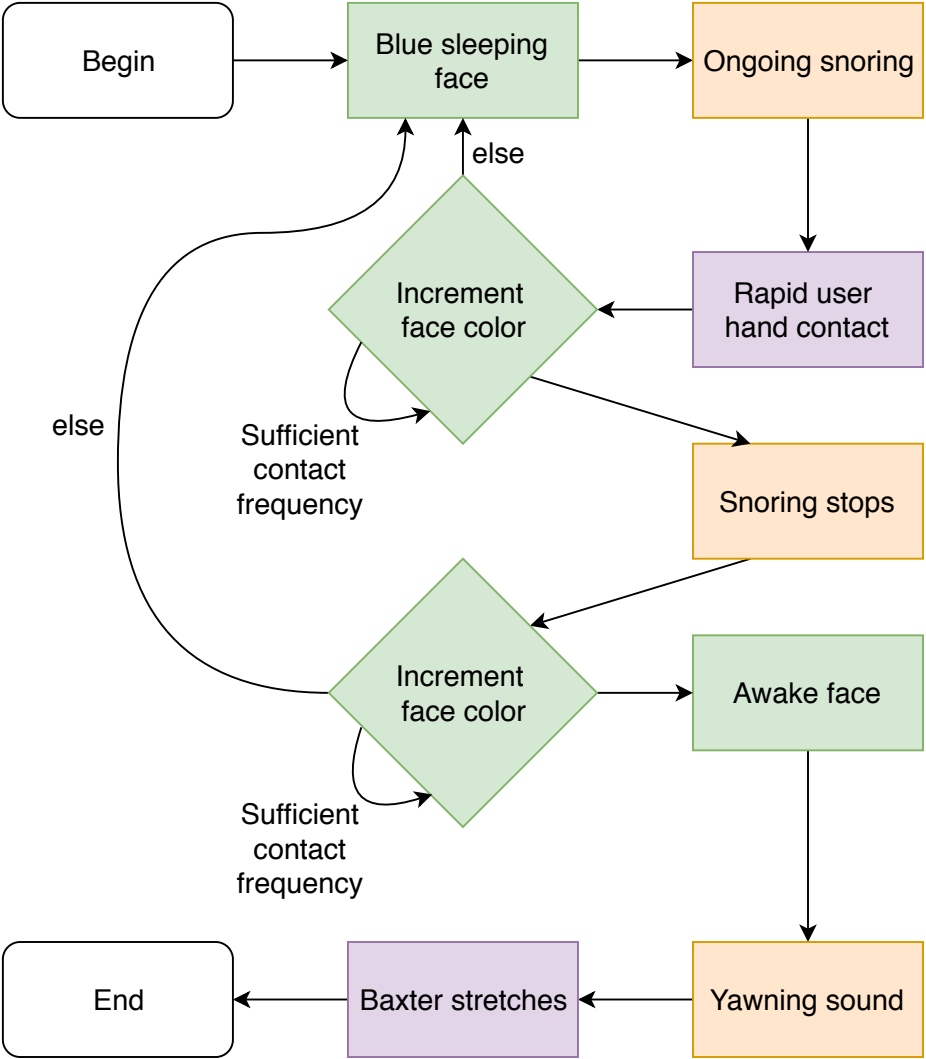

Supplement: Supplementary file 13 — Additional file 7 Gameplay flow of the Agility Game. [file 12984_2020_642_MOESM7_ESM.pdf]

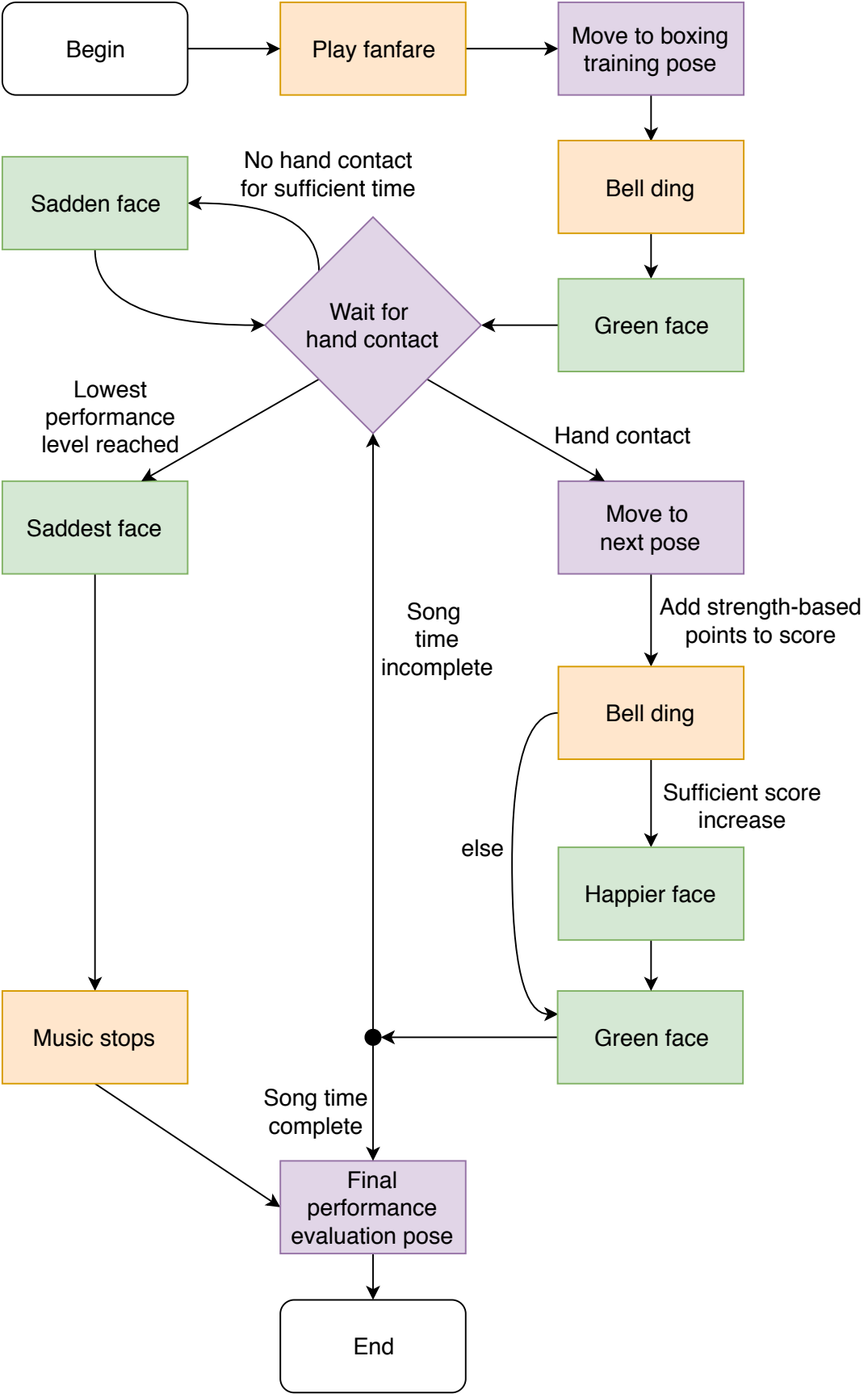

Supplement: Supplementary file 14 — Additional file 9 Gameplay flow of the Strength Game. [file 12984_2020_642_MOESM9_ESM.pdf]

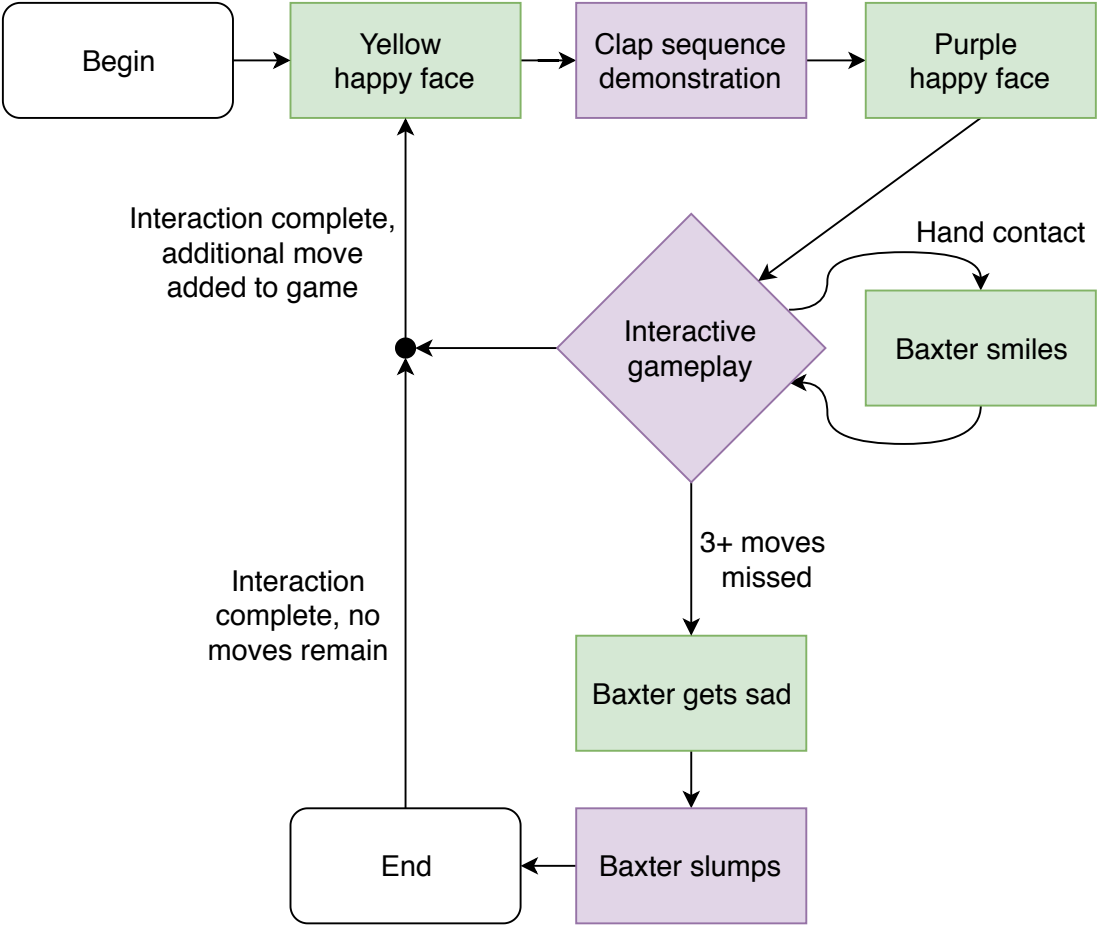

Supplement: Supplementary file 15 — Additional file 11 Gameplay flow of the Handclap Game. [file 12984_2020_642_MOESM11_ESM.pdf]

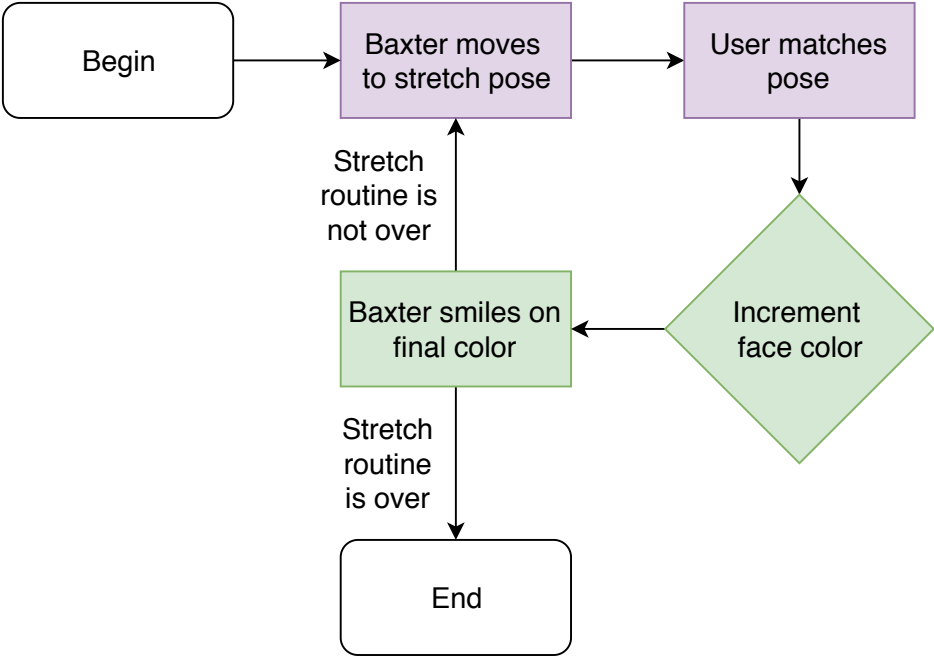

Supplement: Supplementary file 16 — Additional file 13 Gameplay flow of the Roboga Game. [file 12984_2020_642_MOESM13_ESM.pdf]

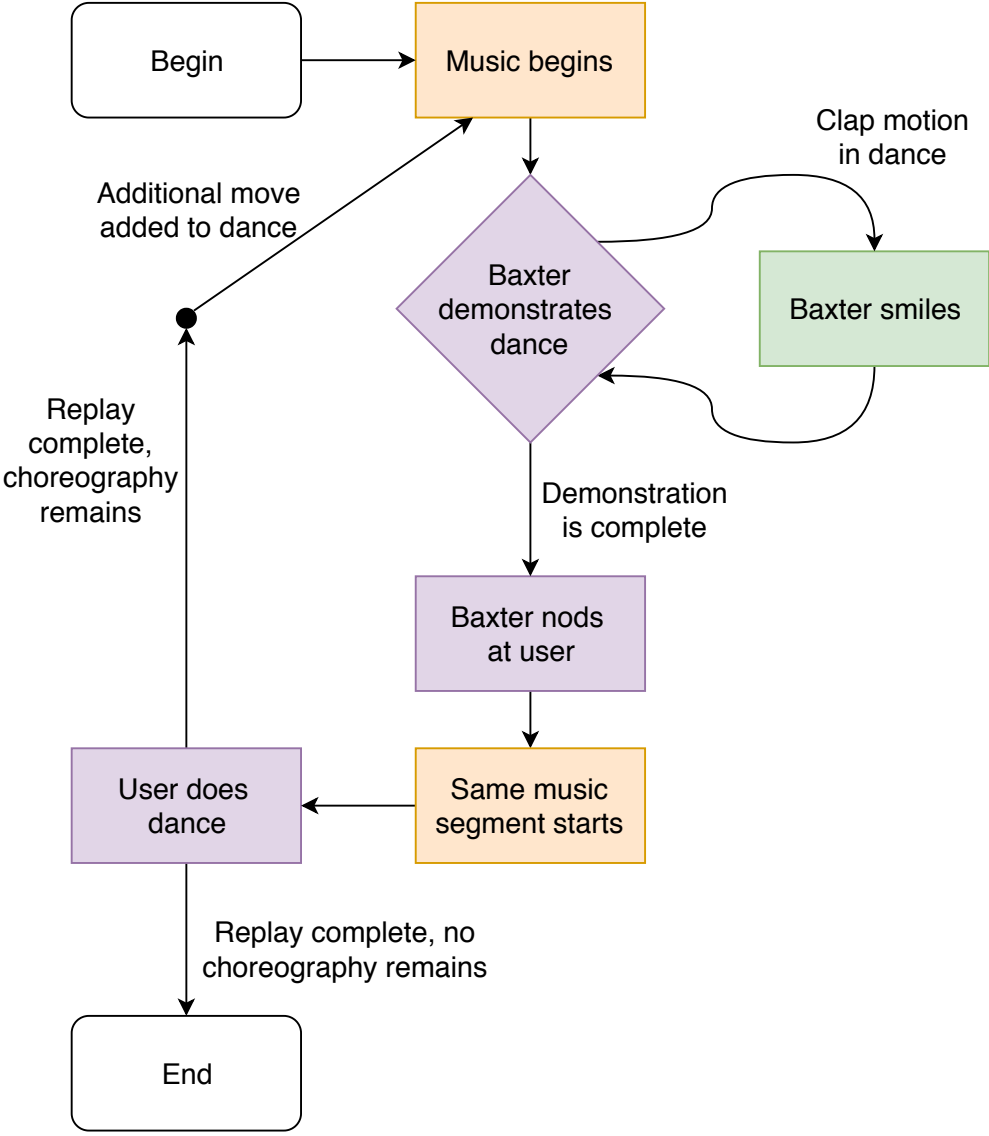

Supplement: Supplementary file 17 — Additional file 15 Gameplay flow of the Flamenco Game. [file 12984_2020_642_MOESM15_ESM.pdf]
