# Supplementary material for: Exercising with Baxter: preliminary support for assistive social-physical human-robot interaction
Source: J Neuroeng Rehabil. 2020 Feb 17;17:19. doi: 10.1186/s12984-020-0642-5 (PMC7027056; doi:10.1186/s12984-020-0642-5)
Supplement: Supplementary file 18 — Additional file 18 Opening survey (survey 1) completed by study participants. [file 12984_2020_642_MOESM18_ESM.pdf]

Opening Evaluation

Subject number ________________

Please rate how much you agree or disagree with each of the following statements by marking your response on each axis with an “x”:

I think using the robot is a good idea.

|____|

Strongly Disagree Strongly Agree

I am afraid to make mistakes while using the robot.

|____|

Strongly Disagree Strongly Agree

I am afraid to break something while using the robot.

|____|

Strongly Disagree Strongly Agree

People would be impressed if I had such a robot.

|____|

Strongly Disagree Strongly Agree

Robots are nice to work with.

|____|

Strongly Disagree Strongly Agree

I am afraid that I could lose my job because of a robot.

|____|

Strongly Disagree Strongly Agree

I could cooperate with the robot.

|____|

Strongly Disagree Strongly Agree

I think the robot is easy to use.

|____|

Strongly Disagree Strongly Agree

I like the presence of the robot.

|____|

Strongly Disagree Strongly Agree

I could do activities with this robot.

|____|

Strongly Disagree Strongly Agree

I feel threatened by the robot.

|____|

Strongly Disagree Strongly Agree

This robot would be useful for me.

|____|

Strongly Disagree Strongly Agree

This robot could help me.

|____|

Strongly Disagree Strongly Agree

This robot could support me.

|____|

Strongly Disagree Strongly Agree

I consider the robot to be a social agent.

|____|

Strongly Disagree Strongly Agree

I feel understood by the robot.

|____|

Strongly Disagree Strongly Agree

I feel comfortable while interacting with the robot.

|____|

Strongly Disagree Strongly Agree

I could work with the robot, if someone helped me.

|____|

Strongly Disagree Strongly Agree

I could work with the robot without any help.

|____|

Strongly Disagree Strongly Agree

I could work with the robot, if I had good initial training.

|____|

Strongly Disagree Strongly Agree

I trust the robot.

|____|

Strongly Disagree Strongly Agree

I would follow the example of the robot.

|____|

Strongly Disagree Strongly Agree
