# Supplementary material for: Exercising with Baxter: preliminary support for assistive social-physical human-robot interaction
Source: J Neuroeng Rehabil. 2020 Feb 17;17:19. doi: 10.1186/s12984-020-0642-5 (PMC7027056; doi:10.1186/s12984-020-0642-5)
Supplement: Supplementary file 19 — Additional file 19 Post-game survey (survey 2) completed by study participants. [file 12984_2020_642_MOESM19_ESM.pdf]

Game Survey

Subject number ________________

Game number __________________

During this game, how hard were you exercising?

|____|

Not at All Maximal Amount

(On this scale, a low rating would represent very very light exercise and a high rating would represent very very hard exercise, with many levels in between)

Please rate your feelings while playing this game on the following scales:

|____|

Unhappy/Sad Happy/Elated

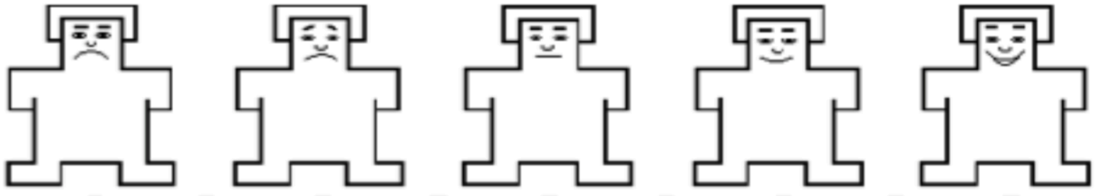

|____|

Calm/Bored Stimulated/Involved

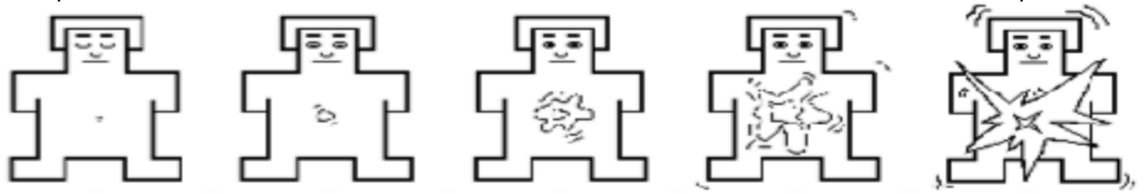

|____|

Not in Control In Control/Dominant

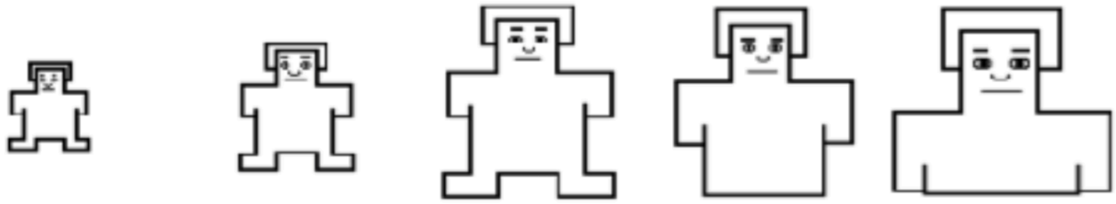

How much pain did you experience while playing this game?

|____|

No Pain Most Severe Pain Imaginable

(On this scale, the lowest rating would reflect no pain, a rating in the leftmost region would reflect mild pain, a rating in the middle region would reflect moderate pain, and a rating in the rightmost region would reflect severe pain. The corresponding pain face scale below provides another pain level representation.)

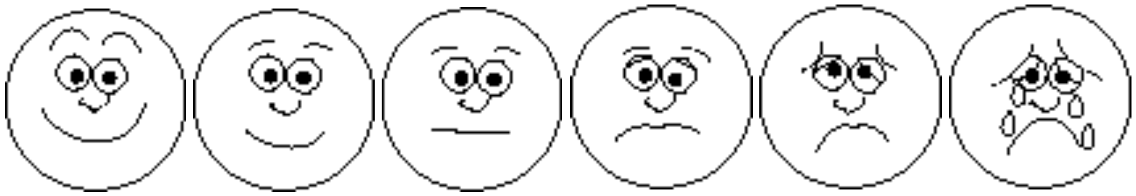

How safe did this robot behavior seem?

|____|

Very Unsafe Very Safe

How much did you enjoy this set of interactions?

|____|

Very Little Very Much

How engaged did you feel throughout this set of interactions?

|____|

Very Unengaged Very Engaged

How well did you perform during this set of interactions?

|____|

Very Badly Very Well

How well did the robot perform during this set of interactions?

|____|

Very Badly Very Well

How rushed did you feel during this set of interactions?

|____|

Very Rushed Not at All Rushed

How calm did you feel during this set of interactions?

|____|

Not at All Calm Very Calm

What comments do you have about this interaction with the robot?

____

____

____

____
