# Supplementary material for: Exercising with Baxter: preliminary support for assistive social-physical human-robot interaction
Source: J Neuroeng Rehabil. 2020 Feb 17;17:19. doi: 10.1186/s12984-020-0642-5 (PMC7027056; doi:10.1186/s12984-020-0642-5)
Supplement: Supplementary file 21 — Additional file 21 Demographic survey (survey 4) completed by study participants. [file 12984_2020_642_MOESM21_ESM.pdf]

Demographic Information

Subject number ________________

Group

☐ A ☐ B

How old are you? ___________________________________

What is your birthdate? (day, month, and year) _________________________________

What is your gender?

☐ Male ☐ Female ☐ Other

What is your handedness?

☐ Left-handed ☐ Right-handed ☐ Ambidextrous (please elaborate: _____________)

What is your profession? ___________________________

Is your education/background technical?

☐ Yes ☐ No

What is your experience level with robots? (please mark response with an “x”)

|____|

No Experience Expert-Level Experience

What is your experience level with Baxter? (please mark response with an “x”)

|____|

No Experience Expert-Level Experience

What is your hometown (city, state, country)? ______________________________
